# On the Mechanism of Cocrystal Mechanochemical Reaction via Low Melting Eutectic: A Time-Resolved In Situ Monitoring Investigation

**DOI:** 10.1021/acs.cgd.2c00262

**Published:** 2022-06-01

**Authors:** Paolo P. Mazzeo, Michele Prencipe, Torvid Feiler, Franziska Emmerling, Alessia Bacchi

**Affiliations:** † Department of Chemistry, Life Sciences and Environmental Sustainability, University of Parma, Parco Area delle Scienze 17/A, 43124 Parma, Italy; ‡ Biopharmanet-TEC, University of Parma, Parco Area delle Scienze 27/A, 43124 Parma, Italy; § BAM Federal Institute for Materials Research and Testing, Richard-Willstätter-Straße 11, D-12489 Berlin, Germany

## Abstract

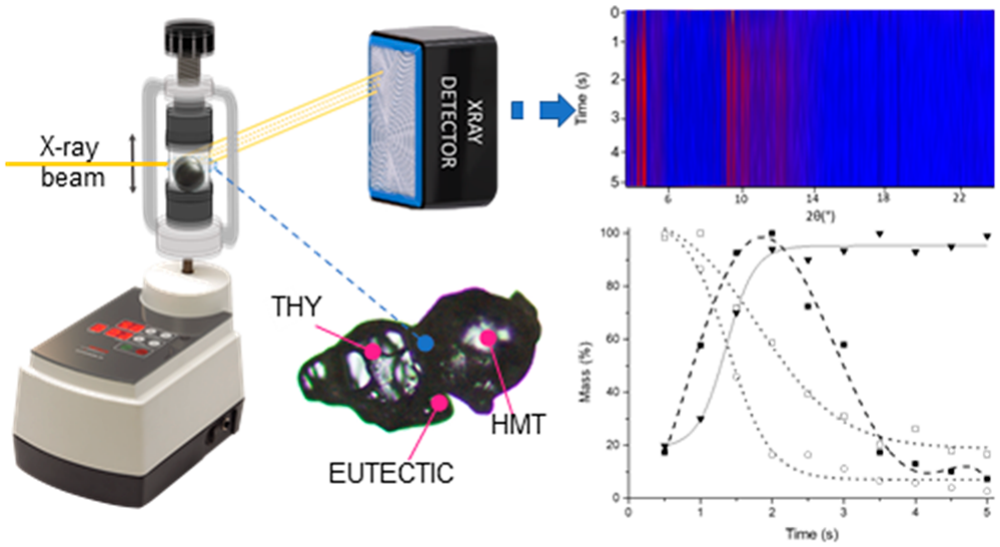

Mechanochemistry has become a sustainable and attractive cost-effective synthetic technique, largely used within the frame of crystal engineering. Cocrystals, namely, crystalline compounds made of different chemical entities within the same crystal structure, are typically synthesized in bulk via mechanochemistry; however, whereas the macroscopic aspects of grinding are becoming clear, the fundamental principles that underlie mechanochemical cocrystallization at the microscopic level remain poorly understood. Time-resolved in situ (TRIS) monitoring approaches have opened the door to exceptional detail regarding mechanochemical reactions. We here report a clear example of cocrystallization between two solid coformers that proceeds through the formation of a metastable low melting binary eutectic phase. The overall cocrystallization process has been monitored by time-resolved in situ (TRIS) synchrotron X-ray powder diffraction with a customized ball milling setup, currently available at μSpot beamline at BESSY-II, Helmholtz-Zentrum Berlin. The binary system and the low melting eutectic phase were further characterized via DSC, HSM, and VT-XRPD.

## Abstract 2

Mechanochemical cocrystallization between thymol and hexamethylenetetramine occurs through the formation of a low melting eutectic (LME) phase. The overall process, going to completion in 5 seconds, is monitored at the subsecond regime by time-resolved in situ (TRIS) synchrotron XRPD with a customized ball milling setup. Binary phase diagram, HSM, and VT-XRPD complete the characterization of the LME.

## Introduction

Mechanochemistry has become popular as a sustainable and cost-effective synthetic technique^1−4^ for the synthesis of different classes of organic^5,6^ and inorganic^7−10^ compounds as well as metal–organic materials.^11−17^ It is increasingly clear that many traditional solution-based chemical reactions can, in principle, be carried out via mechanochemistry with no (or minimal) use of solvent^1,17−21^ and, for this reason, the International Union for Pure and Applied Chemistry (IUPAC) named it as one of ten chemical innovations that would change our world.^22^

Cocrystals are crystalline compounds made of different molecular entities taken together by intermolecular forces within the same crystal structure.^23−27^ Cocrystallization has been largely investigated in the modern literature^28−33^ since the novel intermolecular networks established between the molecular species involved can tune the physical characteristics^24,34,35^ (e.g., solubility, volatility, melting point) of the single molecular entities when in their pure form. A direct correlation of crystal structure/properties is at the basis of the cocrystal design and application of molecular materials.^15,36,37^

Cocrystals are typically synthesized in bulk via mechanochemistry;^18,38−41^ however, whereas the macroscopic aspects of grinding are becoming clear, the fundamental principles that underlie mechanochemical cocrystallization at the microscopic level remain poorly understood.^42^ Despite their evident utility, this lack of comprehension *de facto* inhibits the outbreak of cocrystals that remain confined within the boundaries of the pharmaceutical industry,^43−46^ with the exception of a few examples.^32,39,47−51^

Clearly, the mass transport and reagent diffusion represent the key step of the overall cocrystal formation process. Only a few interpretations reported in the recent literature suggest that the diffusion process can occur through a gas,^52,53^ liquid,^42,54,55^ or amorphous^56^ phase as a function of the coformers used.

Time-resolved in situ (TRIS) monitoring approaches have opened the door to exceptional detail regarding mechanochemical reactions.^57−60^

**Scheme 1:**
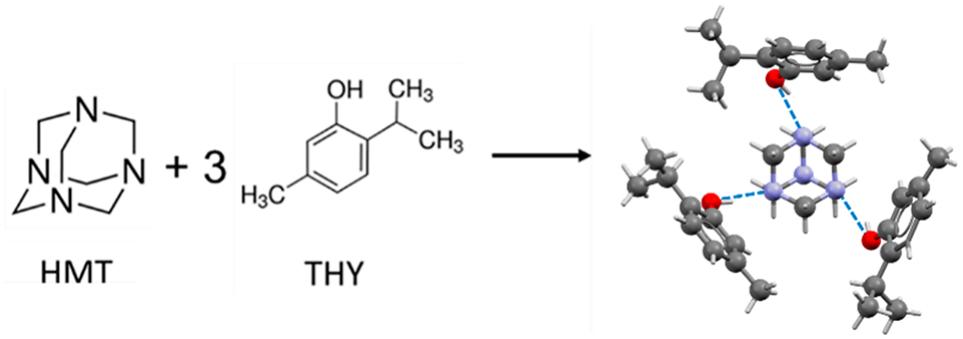
Schematic Representation of the THY:HMT 3:1 Cocrystals Thumbnail image of the corresponding crystalline structures showing the coformers assemblies. All non-H atoms are reported in ball-and-stick style. Color code: C = gray, N = blue, O = red. Hydrogen atoms are reported in capped stick style for the sake of clarity. Blue dashed lines represent the intermolecular H-bonds.

We here report direct evidence of solid–solid cocrystal formation between thymol (an essential oil component extracted from the thyme plants) and hexamethylenetetramine (HMT) that proceeds through the formation of a metastable binary low-melting eutectic (LME) (Scheme 1).

LME is a binary phase composition whose melting point lies below ambient temperature. The formation of a liquid intermediate has a key role in the mass transport of the coformers in solventless cocrystal formation.

The whole mechanochemical process has been monitored by fast time-resolved in situ (TRIS) synchrotron radiation X-ray powder diffraction (XRPD)^57−59,61,62^ at a subsecond data collection frequency, and the low-melting eutectic has been fully characterized by thermal analyses (DSC, HSM, VT-XRPD).

The present cocrystal THY:HMT 3:1 has already been proposed elsewhere within the frame of green pesticides^50^ and food preservative alternatives^51^ with the thymol biologically active against Gram– and Gram+ pathogens.^50,51,63,64^

However, the low water solubility and high volatility of pure thymol intrinsically limit its direct application in the agrochemical and food industry. Cocrystallization has been recently proposed to mitigate its negative performances, thus obtaining a stimuli-responsive material able to tune the release of the essential oils components as a function of the environmental conditions.^50^

## Experimental Section

### Fast Time-Resolved In Situ Monitoring

The ball mill grinding experiments were performed by means of a Fritsch Pulverisette 23 shaker mill with a vertical movement. This mill has a fixed amplitude of 9 mm and adjustable frequency from 15 to 50 Hz with an adjustable timer. A 2.3 mL jar was custom-designed at BAM and consists of three pieces, two stainless steel or polyvinyl chloride (PVC) end pieces and a transparent Perspex middle segment of 0.75 mm thickness. The overall size of the jar is 40 mm with an internal diameter of 12 mm.^60^ X-ray powder diffraction (XRPD) data were collected at μSpot (BESSY-II, Helmholtz Zentrum Berlin) with a low-energy incident beam (17 KeV) of ø 150 μm and an Eiger 9 M 2D detector. Data were collected with an accumulation time of 500 ms per frame while the mill was shaking. Sample-to-detector distance was set at ca. 250 mm. Sequential multiphase Rietveld refinement was performed with TOPAS v 6^65^ to extrapolate the relative amount of the chemical species involved in the mechanochemical reaction.

### Hot Stage Microscopy (HSM)

Cocrystallization of THY and HMT was monitored placing a few crystals (μm order of magnitude) of the two coformers on a glass slide and brought them into contact with a spatula. Different firing profiles (heating and cooling) have been performed by means of a Linkam LTS420 hot stage. The first heating profile was performed by increasing the temperature from 10 to 30 °C at 1 °C min^–1^. The sample was then cooled at 5 °C min^–1^ down to 10 °C and then heated again at 40 °C. The whole process was recorded by means of an Euromex 18MP camera placed on a trinocular optic microscope equipped with a 100× magnification lens.

### Variable Temperature X-ray Powder Diffraction (VT-XRPD)

VT-XRPD measurements of the THY:HMT 3:1 cocrystal were carried out in parallel beam geometry with CuKα radiation on a Rigaku Smartlab XE diffractometer equipped with an Anton-Paar TTK600 nonambient chamber with flat copper sample holder. Data were collected in Bragg–Brentano geometry with the radiation source fix at ω = 4° and the Hypix3000 2D solid-state detector at 2θ = 13°. The solid-state detector was used in 2D mode and still images were collected with an accumulation time of 3 s. Data collection was performed at ambient pressure heating the sample from 20 to 60 °C at 5 °C min^–1^, then cooling it to 10 °C at 5 °C min^–1^ and heating again to 60 °C at 5 °C min^–1^. At the end of the firing profile, the melt sample was slowly thermalized to ambient conditions. Powder patterns were extrapolated integrating the resulting 2D images in the range of 163° < β < 197° to obtain the powder pattern in the range of 5–19° 2θ. Results are reported in Supporting Information Figures 19–24.

### Thermal Analyses

Binary mixtures of THY and HMT were mechanochemically prepared by grinding the coformers at different molar fractions for 30 min at 500 rpm in a Retsch 100 PM planetary ball mill. A 12 mL steel jar was loaded with ca. 300 mg of each mixture and two 9 mm steel ball bearings. Differential scanning calorimetry (DSC) analysis was performed with a PerkinElmer Diamond equipped with a ULSP 90 ultracooler. Thermal analyses were carried out in closed 10 μL Al-pans. All mixtures with χ_HMT_ < 0.33 were exposed to a 20 °C/100 °C/–20 °C/100 °C heating–cooling–heating firing profile. For the mixtures with χ_HMT_ ≥ 0.33, thus with an excess of HMT with respect to the 3:1 cocrystal, a single heating ramp from 20 to 300 °C was performed due to the decomposition process of HMT. All measurements were performed at 5 °C min^–1^ at atmospheric pressure under a constant flow of nitrogen (20 μL min^–1^). The enthalpy of the endothermic or exothermic events, reported in J g^–1^, were determined by integrating the area underneath the thermal peaks.

### Binary Phase Diagram

THY:HMT binary mixtures at different molar ratios were tested to extrapolate the solid/liquid equilibrium curves for THY:HMT 3:1 cocrystal and the single coformers. The thymol liquidus curve was experimentally calculated according to the Schröder–Laar equation,^66^ while the cocrystal liquidus curve was obtained by fitting the experimental data with a second-order polynomial function (see SI for details). Due to the HMT decomposition, the liquidus curve of the HMT could only be approximated.

## Results and Discussion

### Synthesis

The THY:HMT 3:1 cocrystal has been synthesized by grinding the two coformers together in the stoichiometric ratio (THY = 154 mg, HMT = 450 mg). As soon as the two solids were gently bent together, a low melting eutectic formed that became dominant after a few minutes of blending. By grinding the so-formed sticky paste for about 30 min, a whitish solid was obtained. The titled compound was alternatively synthesized by grinding the two coformers in the appropriate stoichiometry in a Retsch 100 PM planetary ball mill for 30 min at 500 rpm. A 12 mL steel jar was loaded with ca. 300 mg and two 9 mm steel ball bearings.

**Figure 1:**
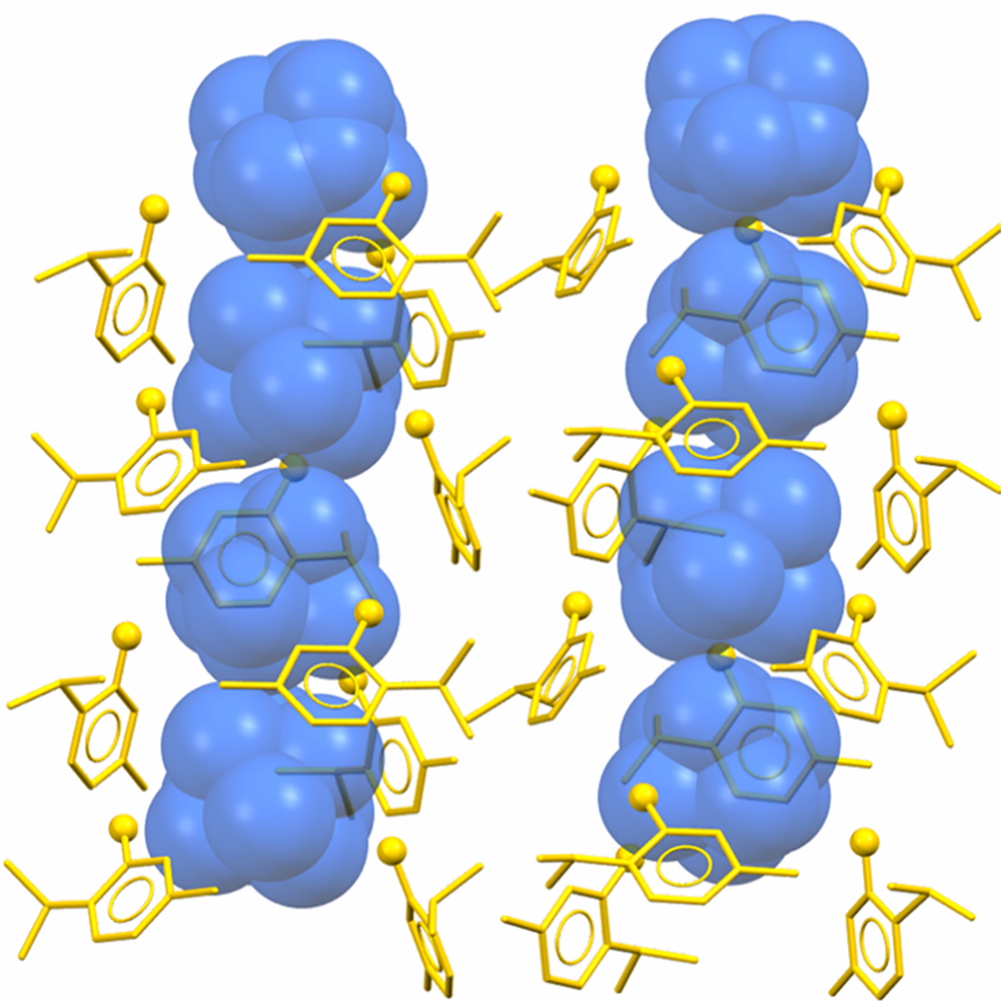
Arrays of THY trimers arranged in columns running along the *a*-axis. HMT is reported in the blue spacefill style, while thymol is shown in the yellow capped stick style. Oxygen atoms in thymol molecules are highlighted in the ball-and-stick style. Hydrogen atoms are removed for the sake of clarity.

The crystal structure of the cocrystal already reported by Mazzeo et al.^50^ consists of supramolecular HMT:THY_3_ aggregates that crystallize in the *P*1̅ space group with a very high molecular multiplicity (Z′ = 4, Z″ = 16). Each independent HMT is hydrogen bonded to three THY molecules in a pseudotrigonal arrangement, thus forming columns of the HMT:THY_3_ aggregates that run along the *a*-axis (Figure 1).

### Time-Resolved In Situ (TRIS) Monitoring

**Figure 2:**
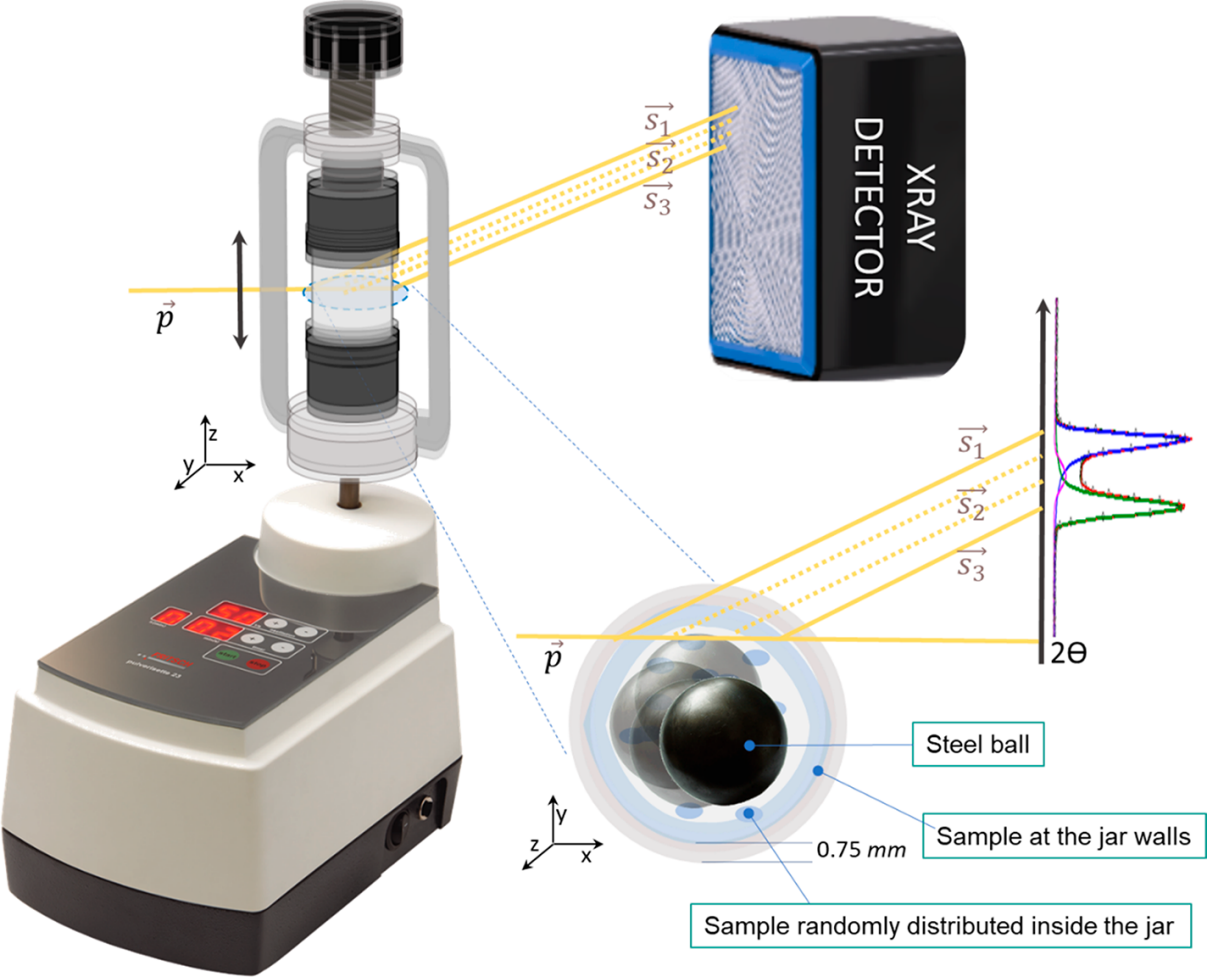
Schematic representation of the milling setup used in this study. The PMMA jars are used with the Fritsch *P*23. The primary X-ray beam *p⃗* (yellow line) passes through the jar and is diffracted by the sample contained within (light blue). Diffraction with this setup results in splitting of each Bragg reflection into a convolution of 2θ positions as the powder inside the jar is distributed across different locations and hence a range of sample-to-detector distances.

**Figure 3:**
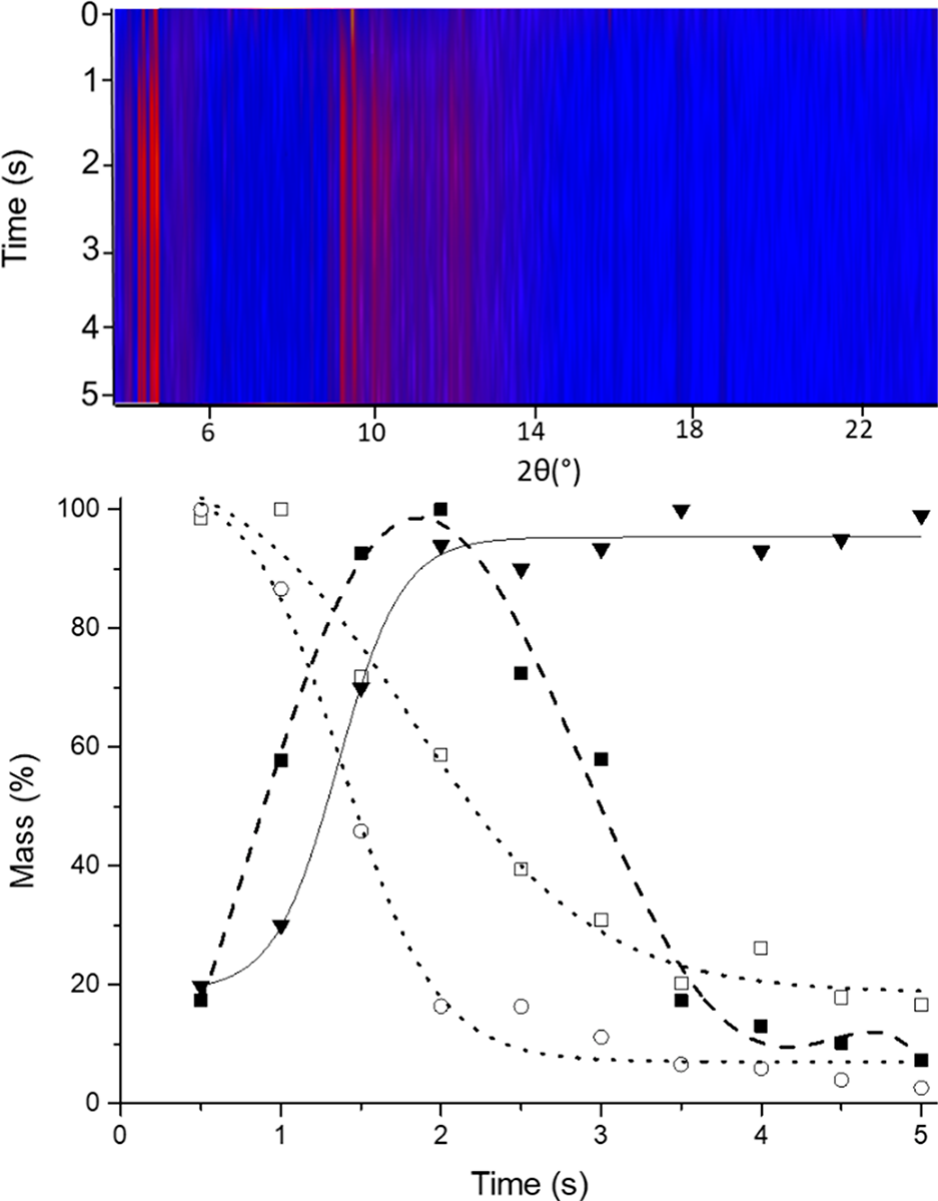
(top) Heatmap plot of XRPD patterns collected as a function of milling time. (bottom) Semiquantitative phase analysis (sQPA) performed via Rietveld Refinement: Experimental data for cocrystal (▼), eutectic phase (■), THY (□), and HMT (○) reported as mass % normalized respect to the maxima of each chemical species. Sigmoidal fit of cocrystal data (solid black line), polynomial fit of the eutectic metastable phase (dashed black line), and sigmoidal fit of coformers (dotted, black line) as a function of the milling time.

The cocrystal synthesis was monitored via TRIS-XRPD with the milling setup recently presented by Lampronti et al.^60^ A Perspex jar with 0.75 mm wall thickness has been loaded with 0.5 mmol of THY and 1.5 mmol of HMT and one 7 mm steel ball bearing (Figure 2). As reported in the Experimental Section, the milling equipment is placed in the synchrotron hutch with the beam passing through the jar. Ideally, the diffraction occurs from a single point, but with the geometry proposed, the beam passes through an elongated sample volume, thereby resulting in a broadening, and ultimately splitting, of the diffracted peaks. XRPD data were collected every 500 ms, while the jar was shaken at 50 Hz. The total conversion occurs in less than 5 s after which only the cocrystal is present. As the coformers are very prone to react at ambient conditions by forming the low melting eutectic binary phase, the jar was loaded at the very last; however, some traces of cocrystal are already present after the first XRPD pattern (Figure 3).

During the milling experiment, it has been demonstrated that part of the milled powder adheres to the jar wall while the remaining powder flows within the jar. This gives rise to the splitting of each measured diffraction peaks into three main components. The inner and outer scattering components arise from powder adhered to the front and back walls of the milling jar, respectively (see  and  in Figure 2), while the scattered intensity between these extremes (see  in Figure 2) arises from powder which flows freely within the jar. The triplet peak shape (Figure 2) was described with three bell-shaped functions: two split-modified Thompson-Cox-Hastings pseudo-Voigt functions for  and , plus one Gaussian function for . The peak displacement caused by each of the scattering vectors , , and , was corrected by modeling the peak positions as reported in Lampronti et al.^60^

A multiphase Rietveld Refinement was performed on the XRPD patterns considering the crystalline phase of each coformer, the cocrystal, and the amorphous phase of the intermediate LME which contributes to the background.

To deconvolute the intrinsic amorphous contribution to the massive extrinsic background due to the Perspex jar, the XRPD pattern of the empty jar was collected in the same experimental conditions and included in the Rietveld Refinement input (see SI for further details).

As reported in Figure 3, the intensity of the single coformer phases monotonically decrease as a function of the milling time. The eutectic phase grew as a metastable intermediate thus increasing in the first part of the milling process and then decreasing to leave the stage to the cocrystal after 5 s from the beginning of the milling process.

### Low Melting Eutectic Characterization

The binary phase diagram confirms that the amorphous quality observed when the two coformers come into contact is indeed the eutectic phase, which is characterized by a melting point below the ambient temperature.

The liquidus curve of THY was obtained using the Schröder–Laar equation^66^ (eq 1) where χ_THY_ is the experimental molar ratio of THY in the mixture under investigation and *T*_THY_ is the melting temperature of thymol as pure component.

Due to the limited number of experimentally accessible data in the narrow range of molar ratio, the cocrystal liquidus curve was obtained with a second-order polynomial fit (eq 2). See SI for further details.
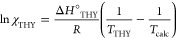
1
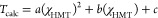
2

**Figure 4:**
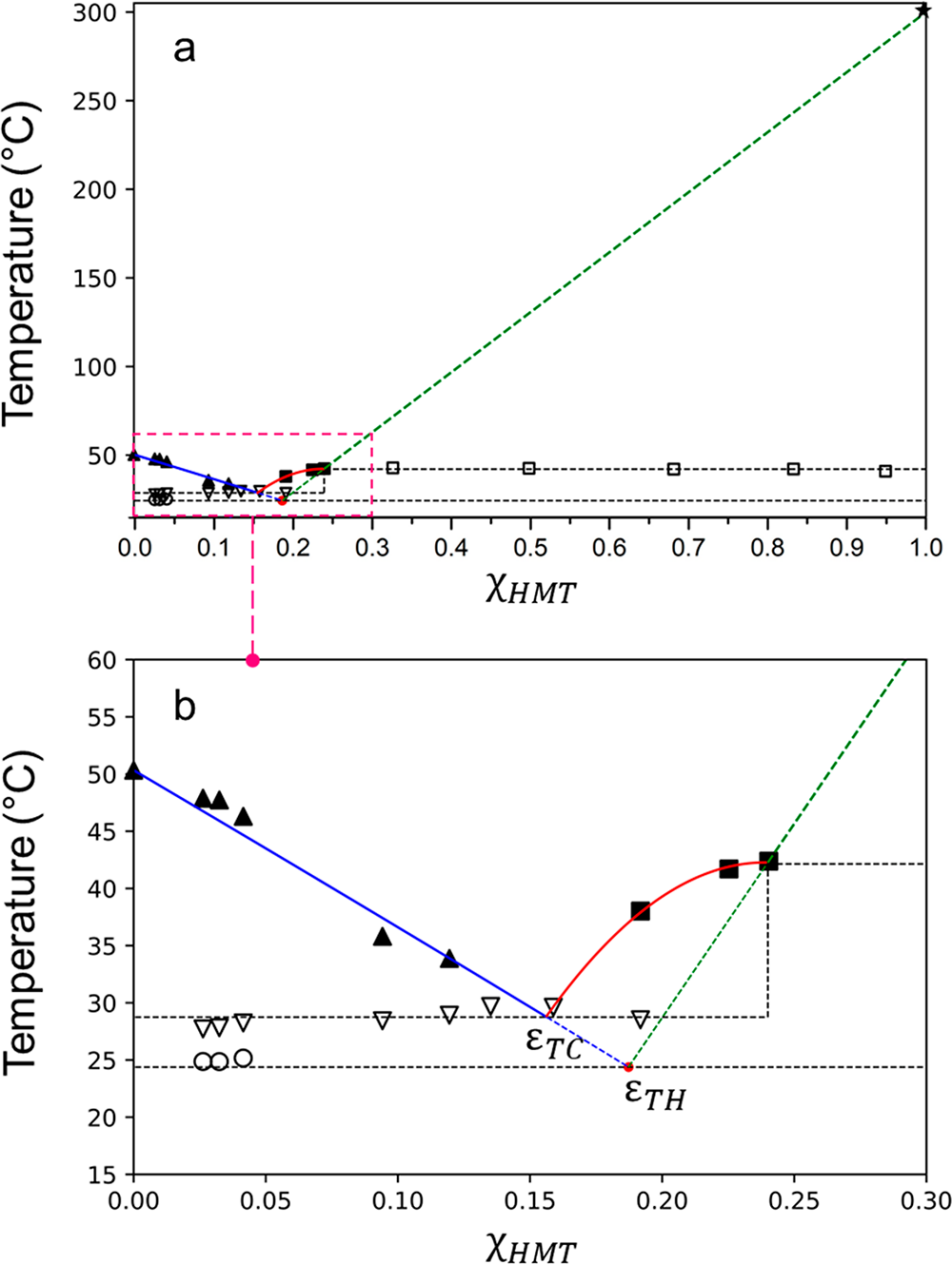
a. Binary solid–liquid phase diagram for the THY/HMT system. Solid lines represent the *liquidus* curves; dashed black lines represent the *solidus* curves. THY, HMT, and cocrystal liquidus curves are depicted in blue, green, and red, respectively. b. Magnified portion of the binary phase in the range of 0 < χ_HMT_ < 0.30 and 15 °C < *T* < 60 °C. The liquidus curves intersect each other at the eutectic composition ε_TC_ (χ_HMT_ = 0.156, *T*_m_ = 28.75 °C) and ε_TH_ (χ_HMT_ = 0.187, *T* = 24.8 °C). Experimental melting points are reported as follows: ▲ = *T*_melt_ of THY residue; ■ = *T*_melt_ of cocrystal residue; ★ = *T*_decomposition_ of HMT; ▽ = *T*_melt_ of ε_TC_; ○ = *T*_melt_ of ε_TH_.

The liquidus curve of HMT was not experimentally derived due to its endothermic decomposition which prevented the accurate evaluation of the melting point of binary mixtures with an excess of HMT (χ_HMT_ > 0.33). A linear fit passing through the pure HMT melting/decomposition and the cocrystal melting represent a first-order approximation of the liquidus curve of HMT. The liquidus curves of THY and HMT intersect at the metastable eutectic compositions ε_TH_, which is characterized by a melting point below ambient temperature in the standard laboratory conditions (*T*ε_TH_ = 24.38 °C) (Figure 4).

**Figure 5:**
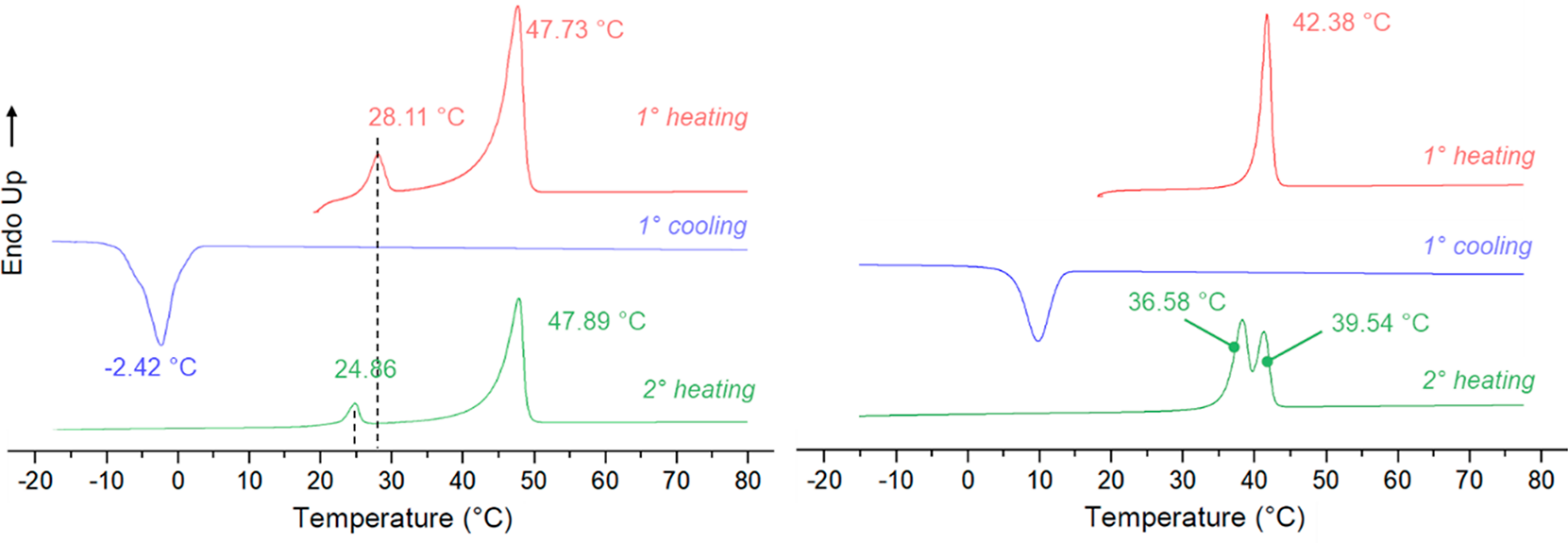
Differential scanning calorimetry traces (endo- up) collected on a binary mixture with χ_HMT_ = 0.026 (a) and χ_HMT_ = 0.240 (b) composition. The firing profile consists of the first heating run from 20 to 80 °C (red line), a cooling run from 80 to −20 °C (blue line) followed by a second heating run from −20 to 80 °C (green line).

As a further proof, the thermal analyses performed on binary mixture with a large excess of THY (χ_HMT_ < 0.04) showed an indented exothermic peak in the cooling run that can be attributed to the concomitant crystallization of the single coformers. The binary eutectic phase thus clearly melts at 24.86 °C in the second heating run as reported in Figure 5, which is consistent with the temperature extrapolated from the binary phase diagram. In Figure 4, the experimental melting point of the LME binary eutectic composition are reported as hole circles.

The *liquidus* curve of THY also intersects the *liquidus* of cocrystal at the eutectic composition ε_TC_ (χ_HMT_ = 0.156, *T*_m_ = 28.73 °C) evidenced by hole triangles in Figure 4. All DSC traces are individually reported in SI.

**Figure 6:**
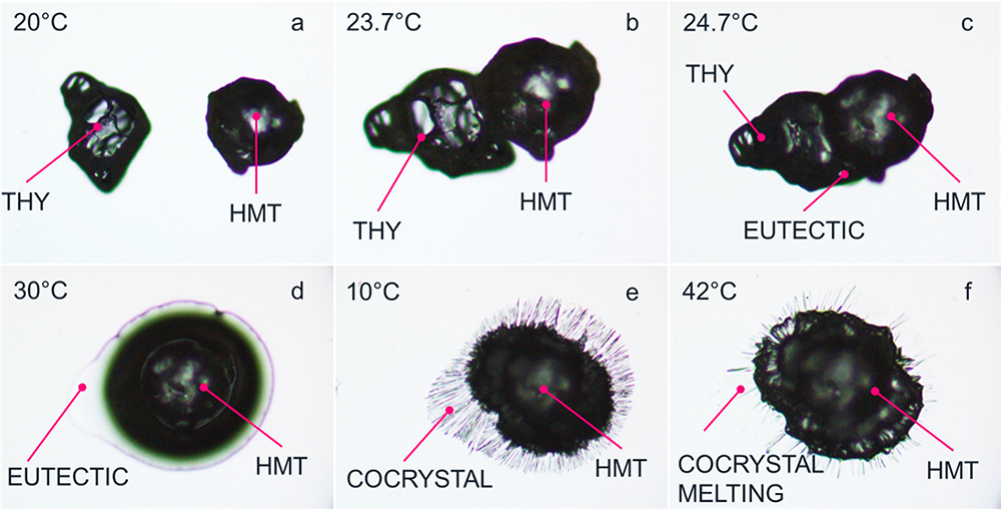
Solid-state reaction between THY and HMT as a function of temperature monitored by HSM. (Mag. 100×) (a) coformers are placed at 20 °C on the glass slide. The crystalline species are solid. (b,c) the THY-HMT eutectic composition starts melting at 23.7 °C. (d) The eutectic is completely melted at 30 °C. A solid residue of coformer is still present. (e) THY:HMT cocrystal crystallized from the eutectic at 10 °C during the cooling process. (f) Cocrystal melts as expected at 42 °C.

The formation of the LME phase as intermediate in the cocrystal formation was additionally described by HSM. A few crystals of THY and HMT (μm order of magnitude) were placed on a glass slide at 10 °C. The temperature was then raised up to 30 °C at 1 °C min^–1^; thus a massive melting of the LME was observed along with a solid residue of the coformers. The sample was then cooled to 10 °C, and a clear crystallization process occurs (Figure 6).

The thermal analysis performed on the cocrystal (χ_HMT_ = 0.25) surprisingly showed in DSC an endothermic event in the second heating of the firing profile at a lower temperature with respect to the first heating run. The thermogram reported in Figure 5 (right, green line) shows two maxima which should be better described as a multistep thermal event consisting of two concomitant endothermic and exothermic processes.

**Figure 7:**
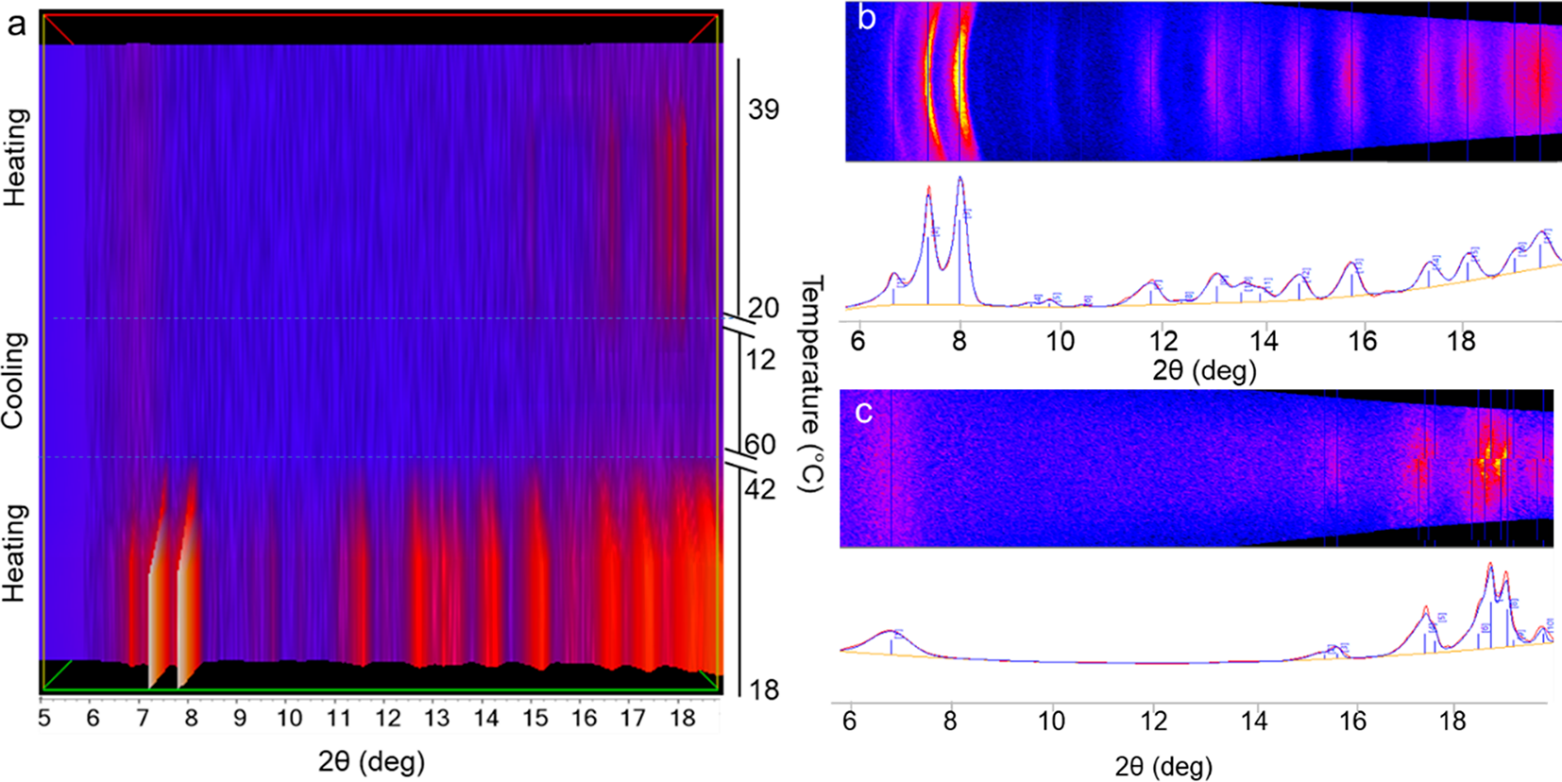
a. Heatmap plot of XRPD patterns extrapolated by integrating the 2D images collected during the VT-XRPD measurement correlated with the acquisition temperature. For the sake of clarity, the temperature scale is not entirely reported, and only relevant events are displayed. b. 2D-XPRD pattern collected at 19.2 °C during the first heating run of the firing profile and its integrated 1D-XRPD pattern corresponding to THY:HMT cocrystal. c. 2D-XPRD pattern collected at 24 °C during the second heating run of the firing profile and its integrated 1D-XRPD pattern corresponding to THY-HMT kinetic phase.

A TRIS-VT-XRPD experiment was then performed to clarify the nature of these thermal events. The THY:HMT 3:1 cocrystal was placed into a nonambient chamber mounted on a laboratory diffractometer (see Experimental Section). 2D data were collected every 3 s, while the sample was heated/cooled in the same firing profile conditions used in the DSC analysis. The cocrystal was the only phase present at the beginning of the firing profile which melted at ca. 42 °C. During the cooling run, a new phase appeared at 12 °C and remained stable during the heating run up to 39 °C when it melted (Figure 7). After melting, the heating was turned off and the sample slowly thermalized to ambient temperature. Single crystals suitable for SCXRD were obtained that confirm the recrystallization of the native THY:HMT cocrystal.

This suggests that the thermal profile influences the formation of a metastable phase that can only be isolated by a kinetically controlled cooling ramp.

## Conclusions

The cocrystallization of thymol and hexamethylenetetramine occurs via solvent-free mechanochemical reaction and proceeds through the formation of a metastable low melting eutectic phase that plays a key role in the mass transport of the coformers. The whole process was monitored via time-resolved in situ X-ray powder diffraction with a customized ball milling setup, currently available at the μSpot beamline at the BESSY-II synchrotron facility. The two coformers react as soon as they are blended, thus forming a low-melting eutectic phase. In the experimental XRPD patterns collected every 500 ms, the intensities of the coformers monotonically decrease, while the background increases as a symptom of the growth of the liquid phase. From the metastable eutectic binary composition, the cocrystallization occurs in less than 5 s. The binary phase diagram suggests that the metastable eutectic phase is indeed characterized by a melting point below ambient temperature, which was further confirmed by hot stage microscopy. A new kinetic phase was observed and isolated through VT-XRPD performed on the binary composition with χ_HMT_ = 0.25, corresponding to the 3:1 THY:HMT stoichiometry.

## Supporting Information Available

Differential scanning calorimetry diagrams, VT-XRPD 2D patterns, Rietveld refinement details (PDF)

## Abbreviations

THY: Thymol
HMT: Hexamethylenetetramine
THY:HMT: Thymol: Hexamethylenetetramine 3:1 cocrystal
LME: Low Melting Eutectic
TRIS: Time-Resolved In Situ
