# Supplementary material for: On the Mechanism of Cocrystal Mechanochemical Reaction via Low Melting Eutectic: A Time-Resolved In Situ Monitoring Investigation
Source: Cryst Growth Des. 2022 Jun 1;22(7):4260–7. doi: 10.1021/acs.cgd.2c00262 (PMC9264353; doi:10.1021/acs.cgd.2c00262)
Supplement: Supplementary file 1 — cg2c00262_si_001.pdf [file cg2c00262_si_001.pdf]

On the mechanism of cocrystal mechanochemical reaction via low melting eutectic: a time-resolved in situ monitoring investigation

*Paolo P. Mazzeo * [a,b], Michele Prencipe [a], Torvid Feiler [c], Franziska Emmerling [c], Alessia Bacchi [a,b]*

a Department of Chemistry, Life Sciences and Environmental Sustainability, University of Parma, Parco Area delle Scienze 17/A, 43124 Parma, Italy. b Biopharmanet-TEC, University of Parma, Parco Area delle Scienze 27/A, 43124 Parma, Italy. c BAM Federal Institute for Materials Research and Testing, Richard-Willstätter-Straße 11, D-12489 Berlin, Germany.

Supporting information

Differential Scanning Calorimetry 2

Binary phase diagram 12

Variable Temperature X-ray Powder Diffraction 13

X-ray Powder Diffraction 17

Rietveld Refinements 17

Differential Scanning Calorimetry

Differential Scanning Calorimetry data were collected for the pure coformers THY and HMT, and for all the mixtures obtained blending the two coformers at different stoichiometries. Besides, each mixture was analysed as soon as it was prepared via ball milling method. Thermograms were recorded and elaborated using Pyris Software V12 (PerkinElmer). The endothermic peaks associated to *solidus* transitions were described reporting the onset temperature while the endothermic peaks associated to *liquidus* transitions were described with the peak temperature. Endothermic and exothermic peaks were integrated and all values are reported in J/g. A summary is reported in table S1.

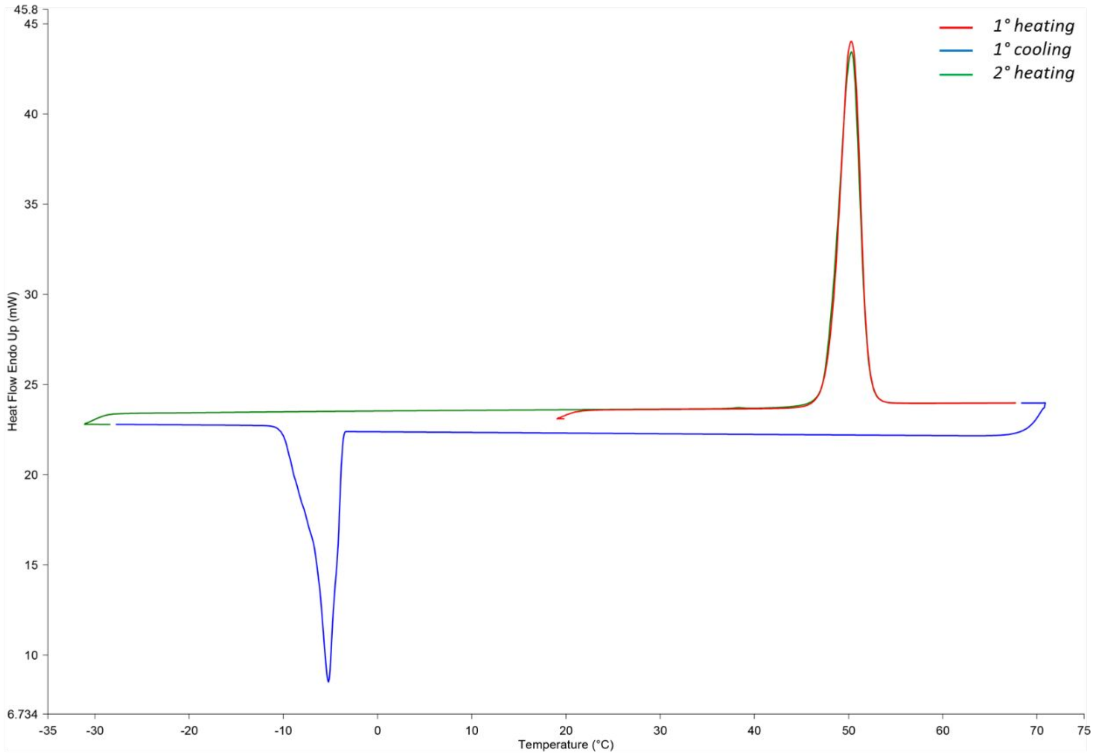

Figure SI 1. Thermogram of pure THY. First heating run from 20 °C to 70 °C (red curve), cooling run from 70 °C to -30 °C (blue curve) and second heating run from -30 °C to 70 °C (green curve). The whole firing profile was performed at 5°C/min.

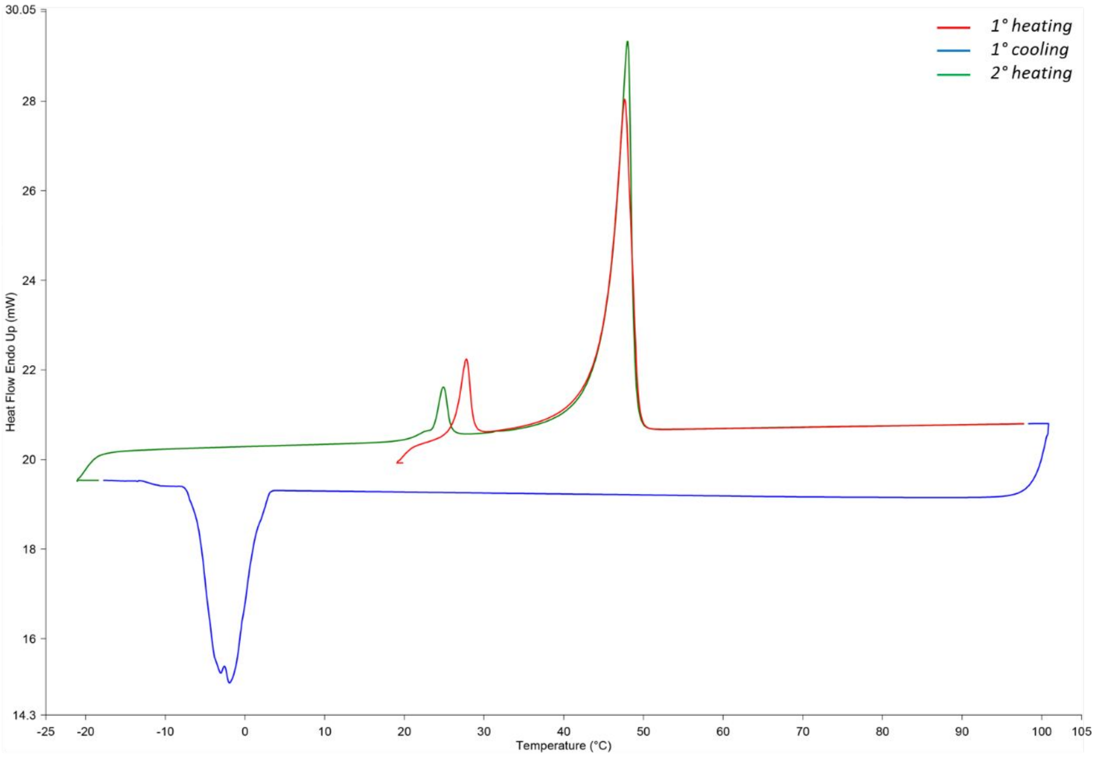

Figure SI 2. Thermogram of THY-HMT binary mixture with χ_{HMT} = 0.026 molar ratio. First heating run from 20 °C to 100 °C (red curve), cooling run from 100 °C to -20 °C (blue curve) and second heating run from -20 °C to 100 °C (green curve). The whole firing profile was performed at 5°C/min.

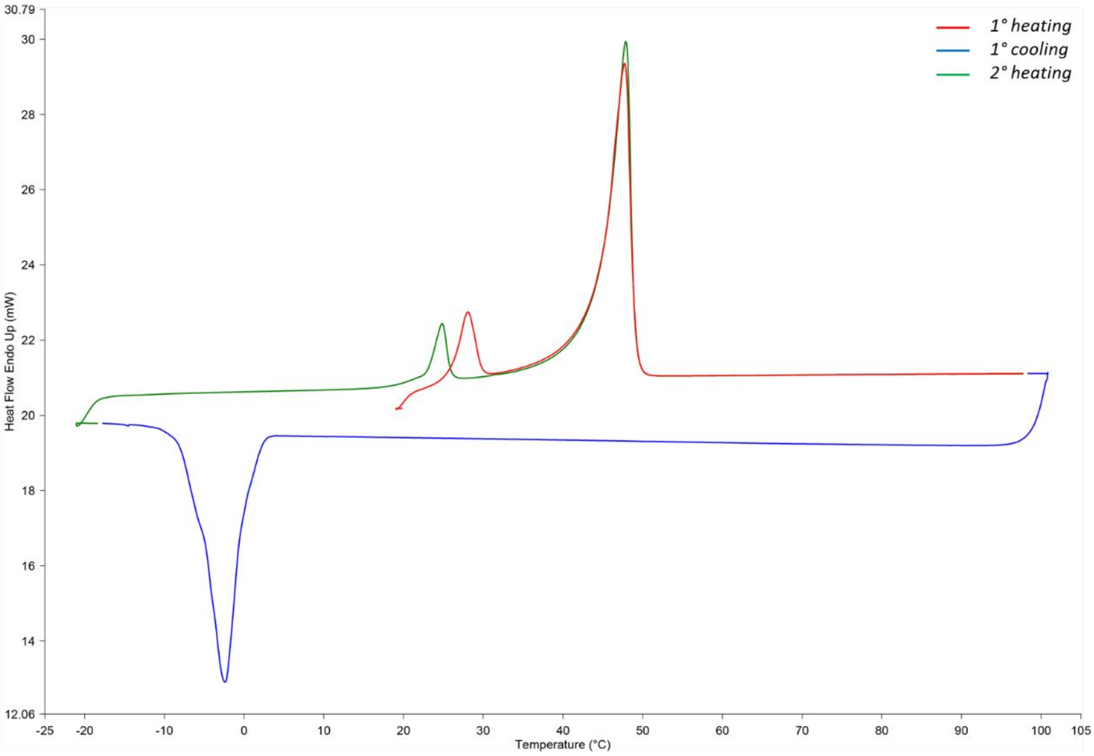

Figure SI 3. Thermogram of THY-HMT binary mixture with χ_{HMT} = 0.032 molar ratio. First heating run from 20 °C to 100 °C (red curve), cooling run from 100 °C to -20 °C (blue curve) and second heating run from -20 °C to 100 °C (green curve). The whole firing profile was performed at 5°C/min.

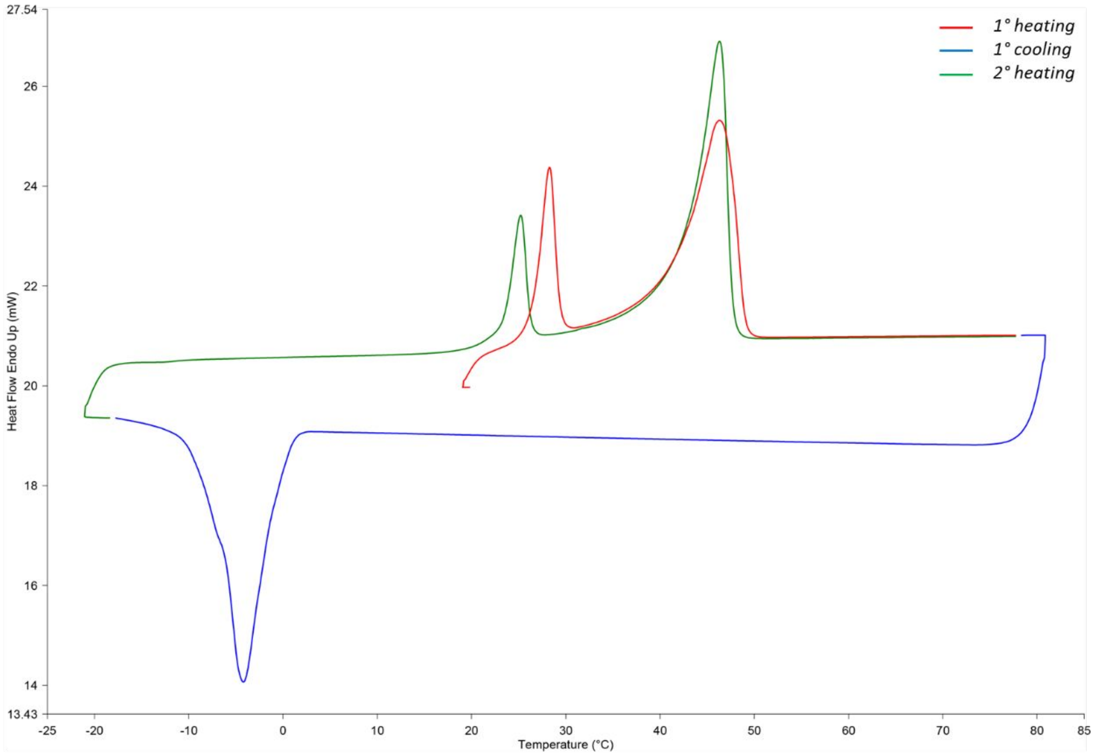

Figure SI 4. Thermogram of THY-HMT binary mixture with χ_{HMT} = 0.048 molar ratio. First heating run from 20 °C to 100 °C (red curve), cooling run from 100 °C to -20 °C (blue curve) and second heating run from -20 °C to 100 °C (green curve). The whole firing profile was performed at 5°C/min.

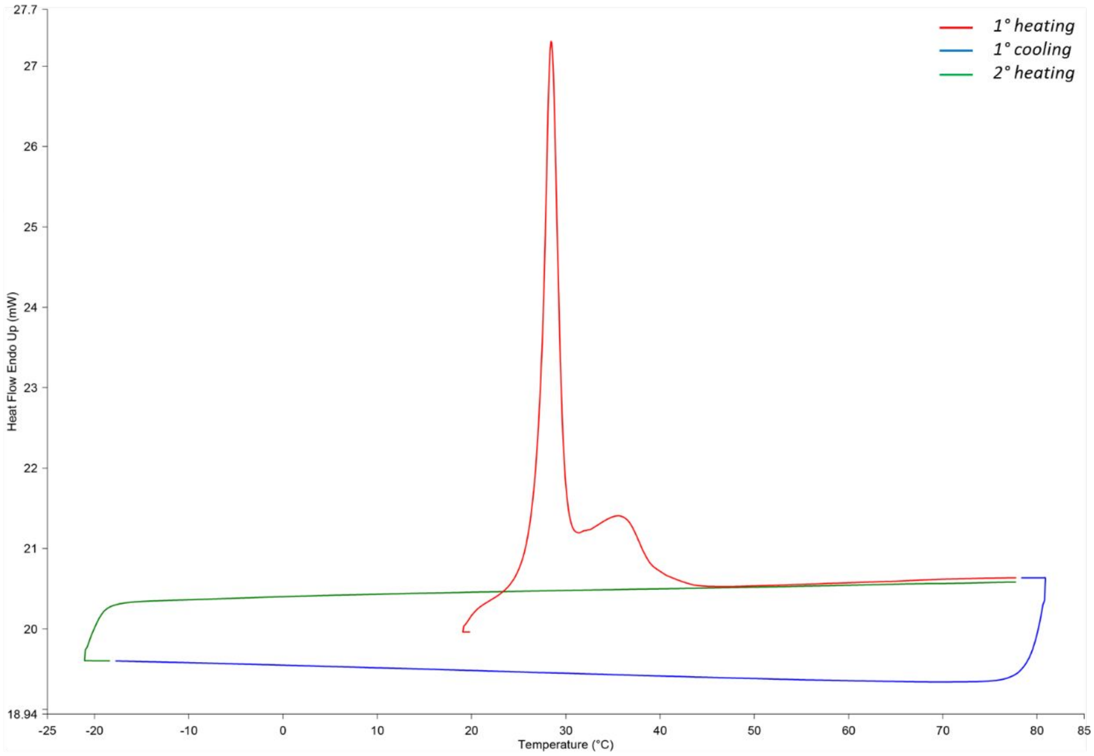

Figure SI 5. Thermogram of THY-HMT binary mixture with χ_{HMT} = 0.113 molar ratio. First heating run from 20 °C to 100 °C (red curve), cooling run from 100 °C to -20 °C (blue curve) and second heating run from -20 °C to 100 °C (green curve). The whole firing profile was performed at 5°C/min.

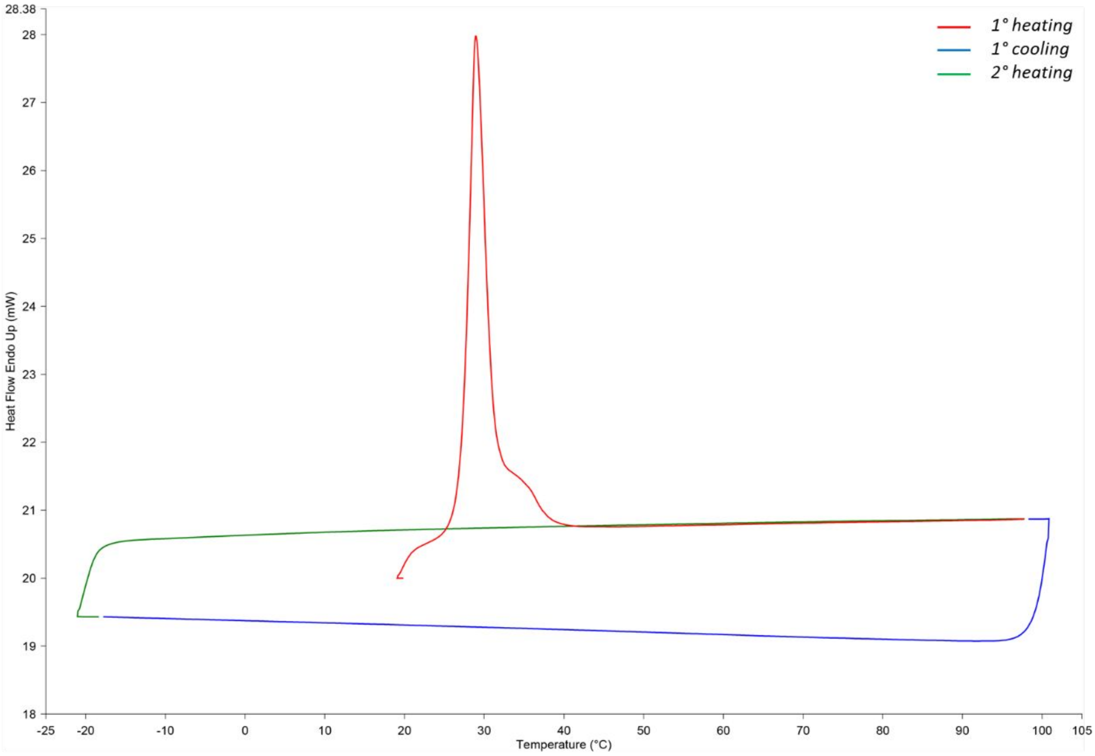

Figure SI 6. Thermogram of THY-HMT binary mixture with χ_{HMT} = 0.131 molar ratio. First heating run from 20 °C to 100 °C (red curve), cooling run from 100 °C to -20 °C (blue curve) and second heating run from -20 °C to 100 °C (green curve). The whole firing profile was performed at 5°C/min.

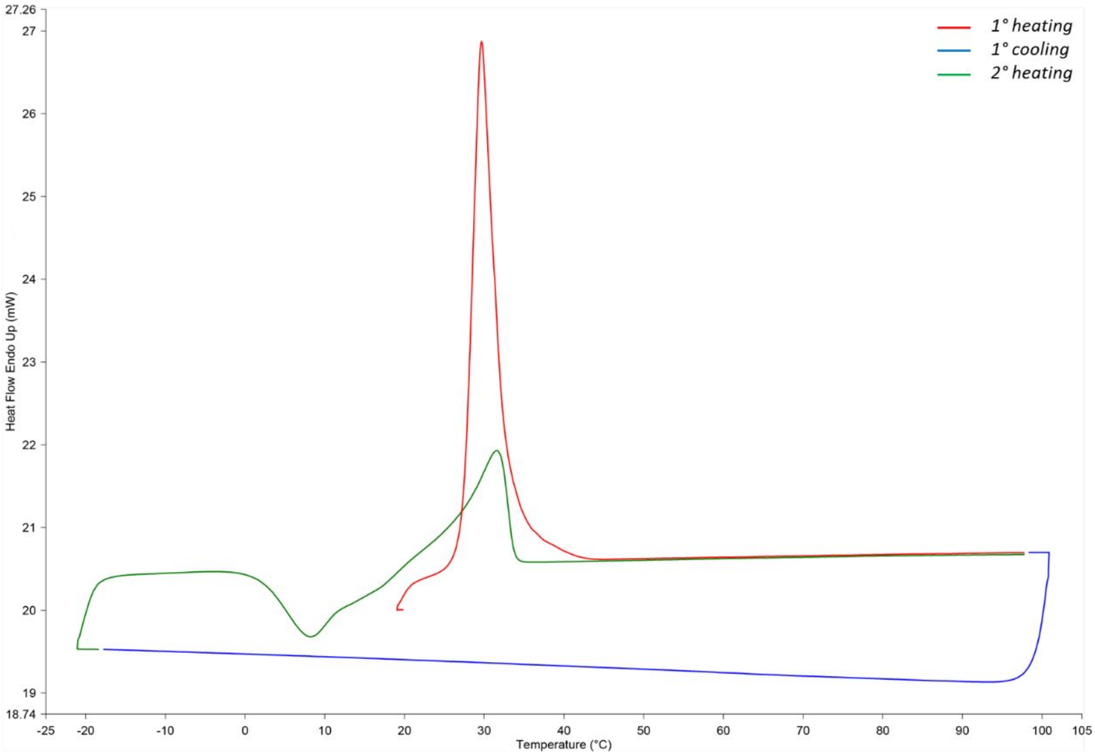

Figure SI 7. Thermogram of THY-HMT binary mixture with χ_{HMT} = 0.135 molar ratio. First heating run from 20 °C to 100 °C (red curve), cooling run from 100 °C to -20 °C (blue curve) and second heating run from -20 °C to 100 °C (green curve). The whole firing profile was performed at 5°C/min.

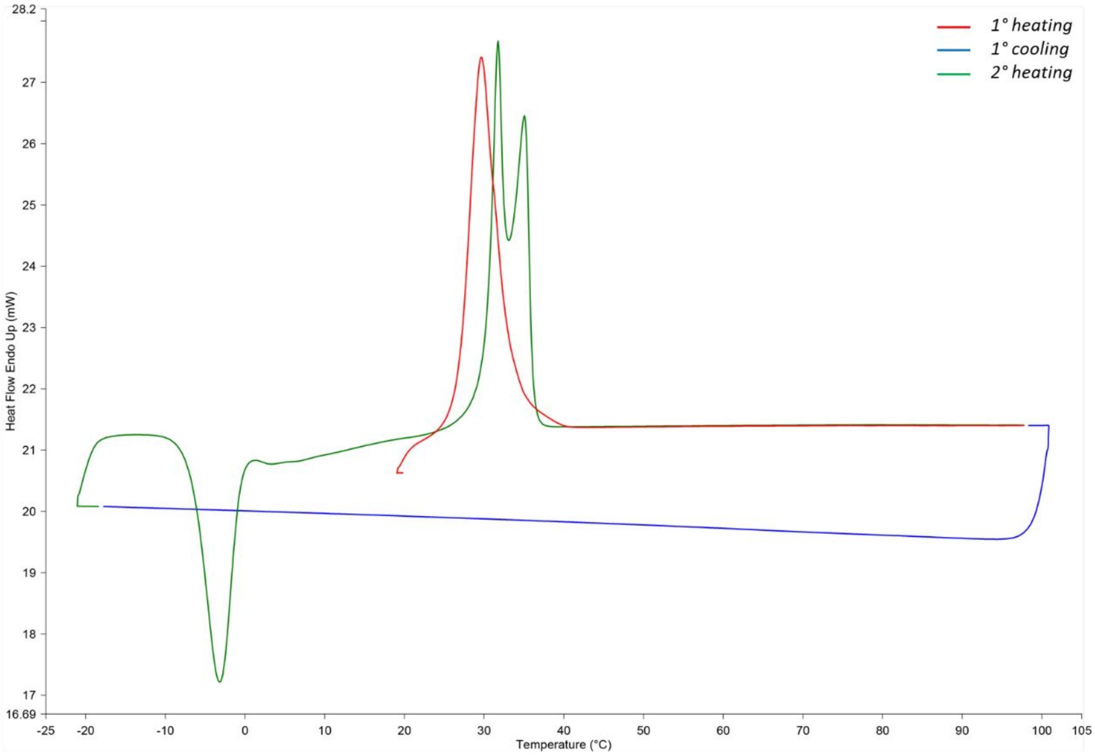

Figure SI 8. Thermogram of THY-HMT binary mixture with χ_{HMT} = 0.159 molar ratio. First heating run from 20 °C to 100 °C (red curve), cooling run from 100 °C to -20 °C (blue curve) and second heating run from -20 °C to 100 °C (green curve). The whole firing profile was performed at 5°C/min.

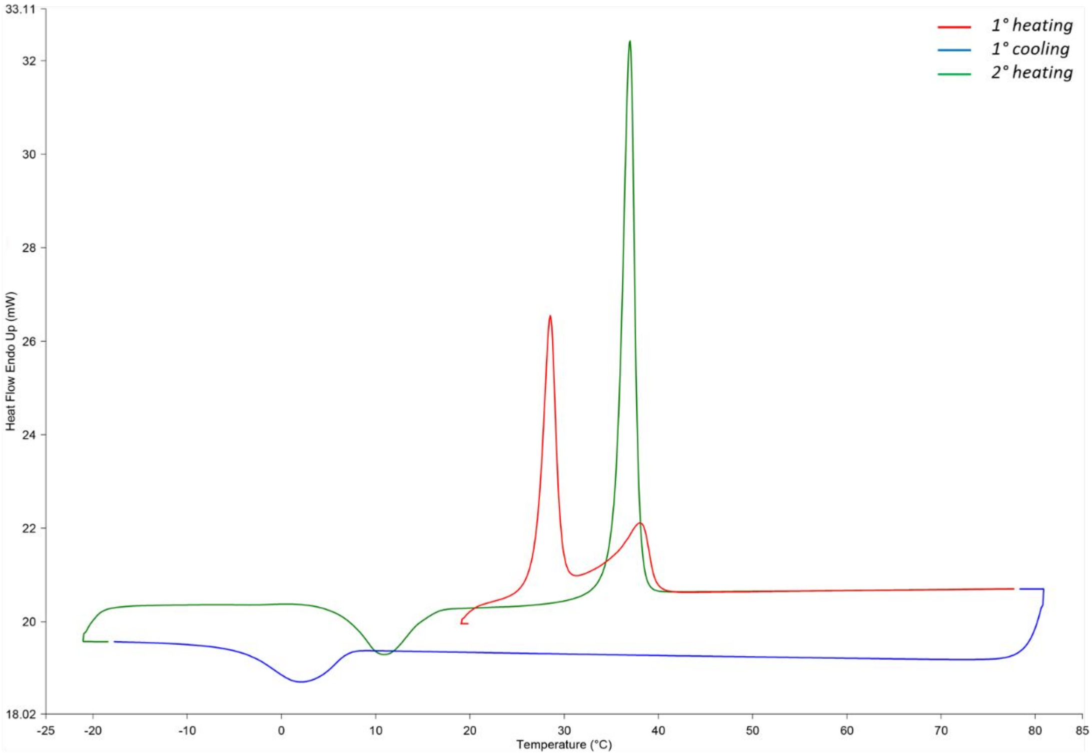

Figure SI 9. Thermogram of THY-HMT binary mixture with χ_{HMT} = 0.192 molar ratio. First heating run from 20 °C to 100 °C (red curve), cooling run from 100 °C to -20 °C (blue curve) and second heating run from -20 °C to 100 °C (green curve). The whole firing profile was performed at 5°C/min.

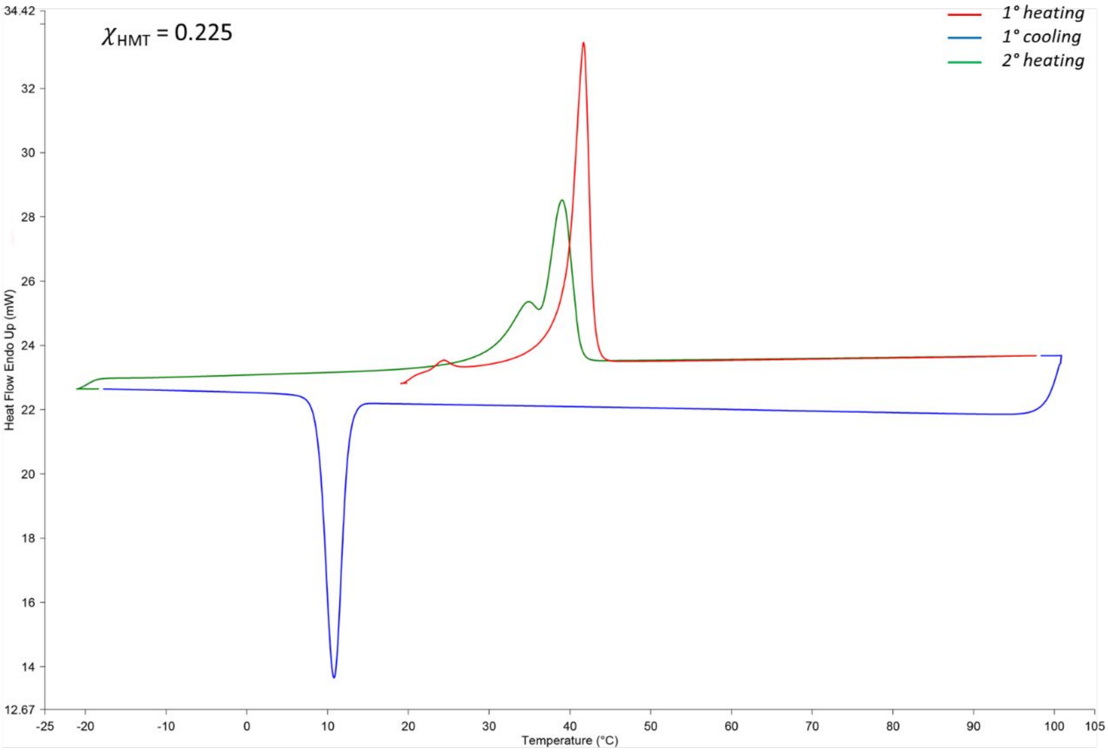

Figure SI 10. Thermogram of THY-HMT binary mixture with χ_{HMT} = 0.225 molar ratio. First heating run from 20 °C to 100 °C (red curve), cooling run from 100 °C to -20 °C (blue curve) and second heating run from -20 °C to 100 °C (green curve). The whole firing profile was performed at 5°C/min.

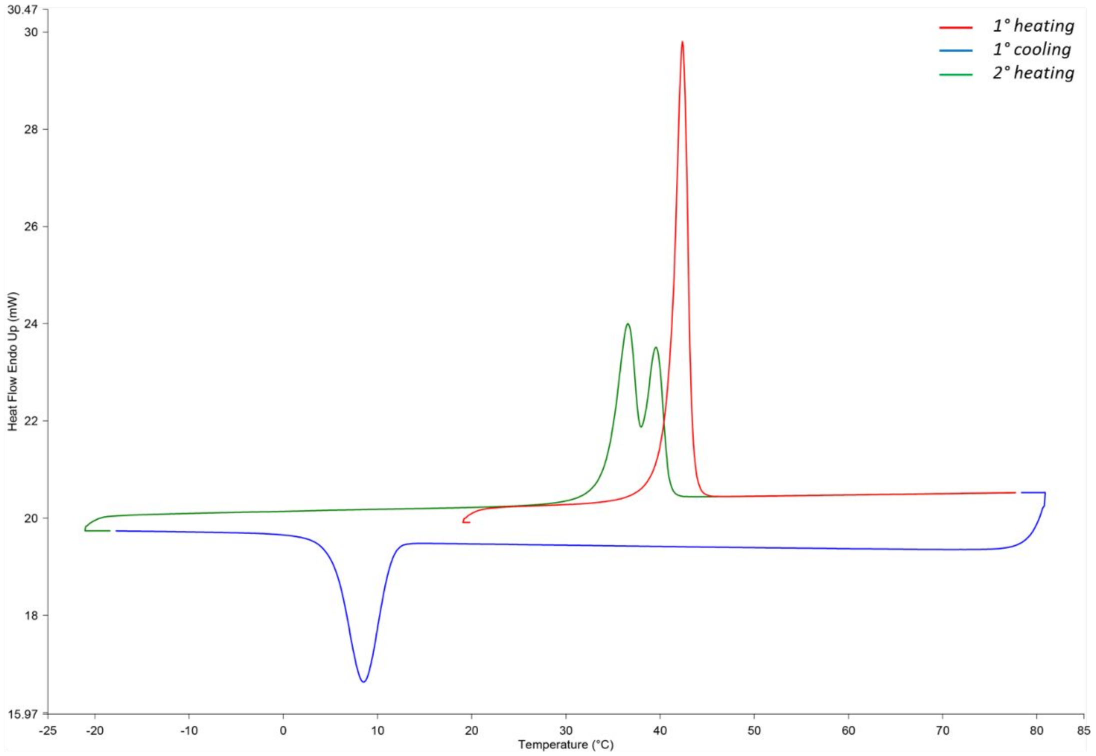

Figure SI 11. Thermogram of THY-HMT binary mixture with χ_{HMT} = 0.240 molar ratio. First heating run from 20 °C to 100 °C (red curve), cooling run from 100 °C to -20 °C (blue curve) and second heating run from -20 °C to 100 °C (green curve). The whole firing profile was performed at 5°C/min.

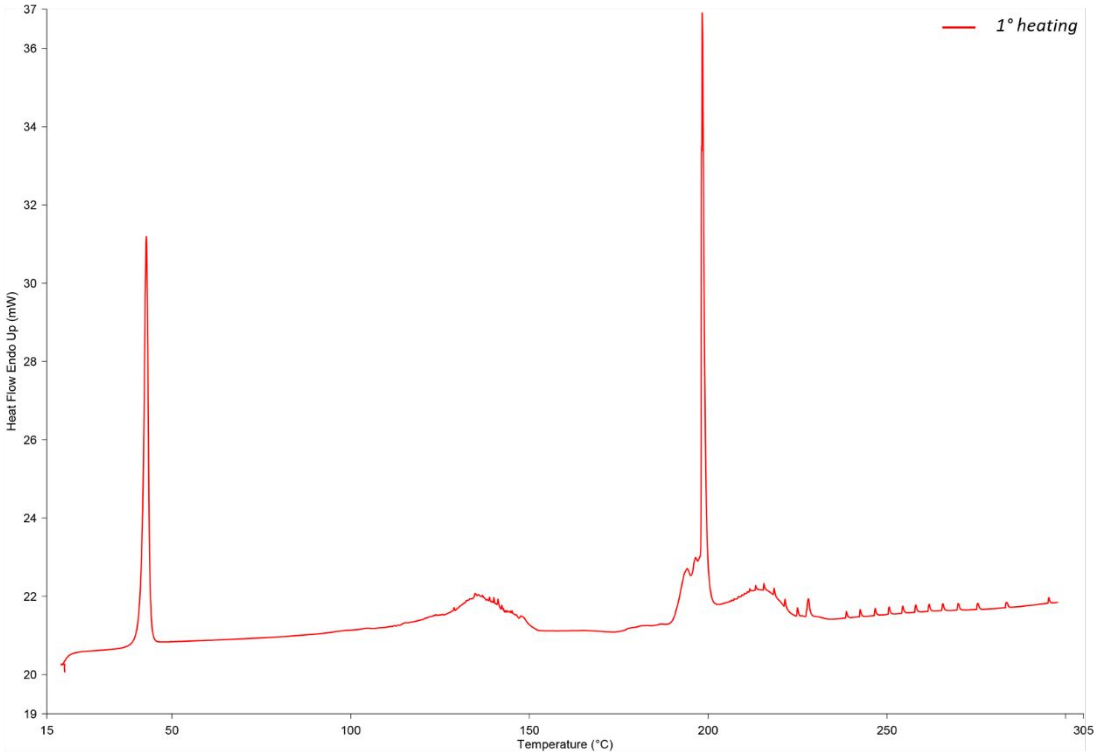

Figure SI 12. Thermogram of THY-HMT binary mixture with χ_{HMT} = 0.327 molar ratio. Heating run from 20 °C to 300 °C at 5 °C/min (red curve). No additional thermal treatments were performed due to since unusual endothermic peaks related to the decomposition of the sample.

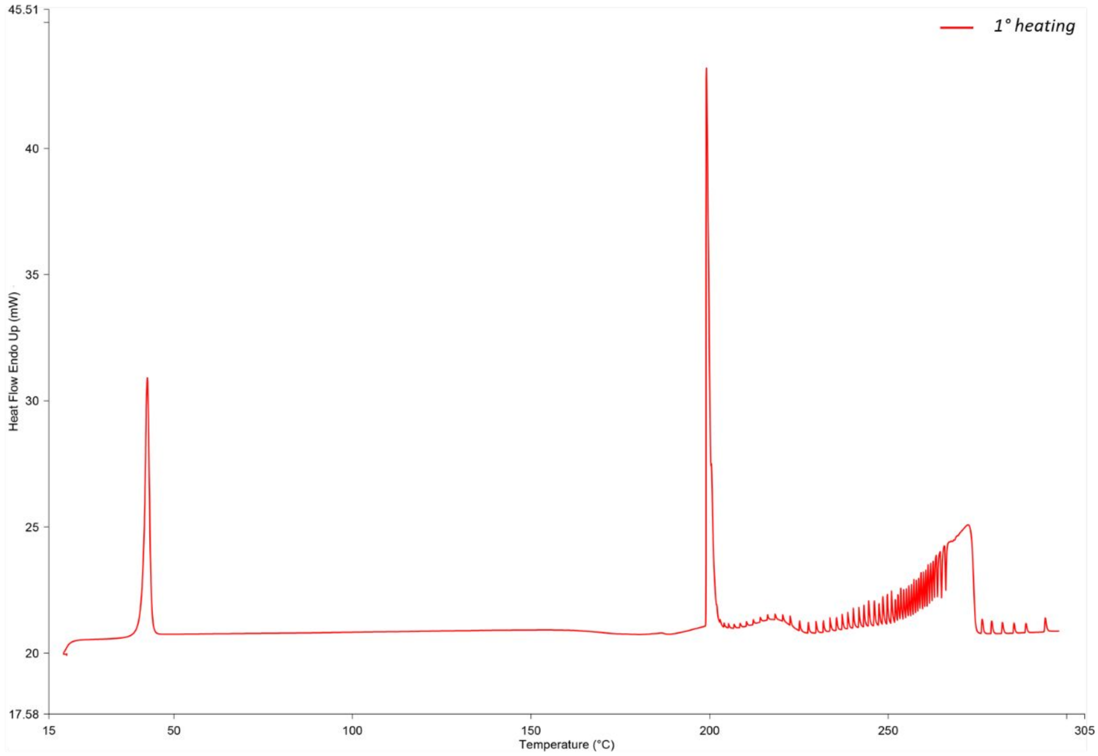

Figure SI 13. Thermogram of THY-HMT binary mixture with χ_{HMT} = 0.500 molar ratio. Heating run from 20 °C to 300 °C at 5 °C/min (red curve). No additional thermal treatments were performed due to since unusual endothermic peaks related to the decomposition of the sample.

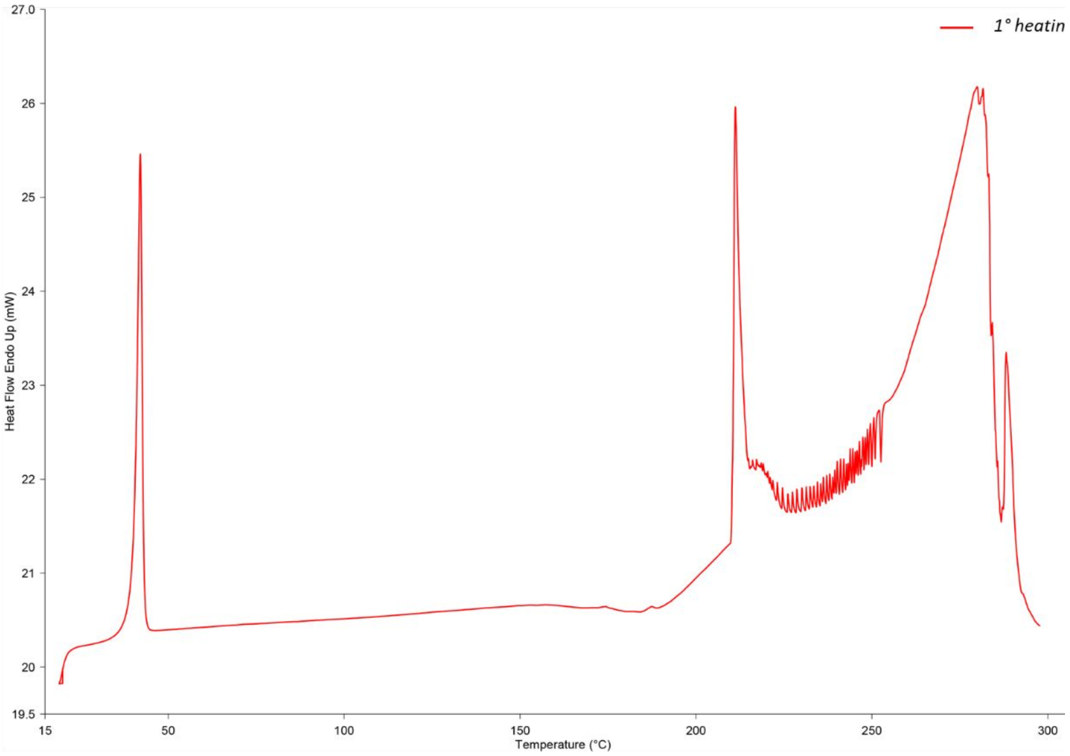

Figure SI 14. Thermogram of THY-HMT binary mixture with χ_{HMT} = 0.681 molar ratio. Heating run from 20 °C to 300 °C at 5 °C/min (red curve). No additional thermal treatments were performed due to since unusual endothermic peaks related to the decomposition of the sample.

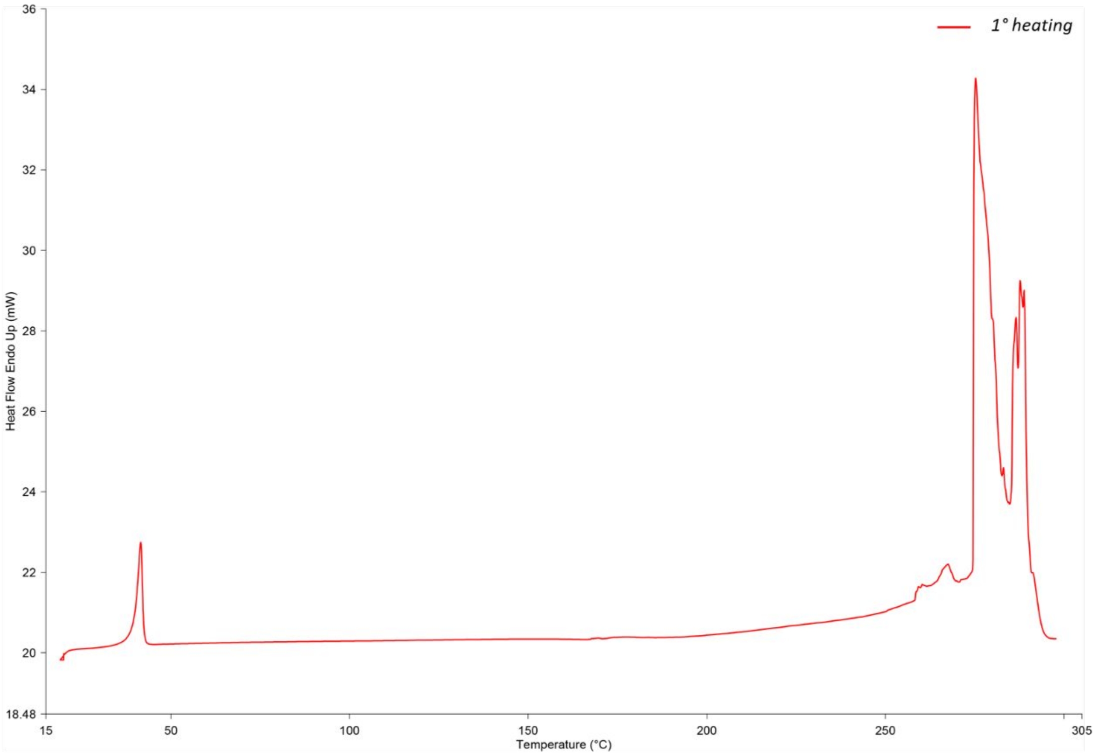

Figure SI 15. Thermogram of THY-HMT binary mixture with χ_{HMT} = 0.832 molar ratio. Heating run from 20 °C to 300 °C at 5 °C/min. No additional thermal treatments were performed due to since unusual endothermic peaks related to the decomposition of the sample.

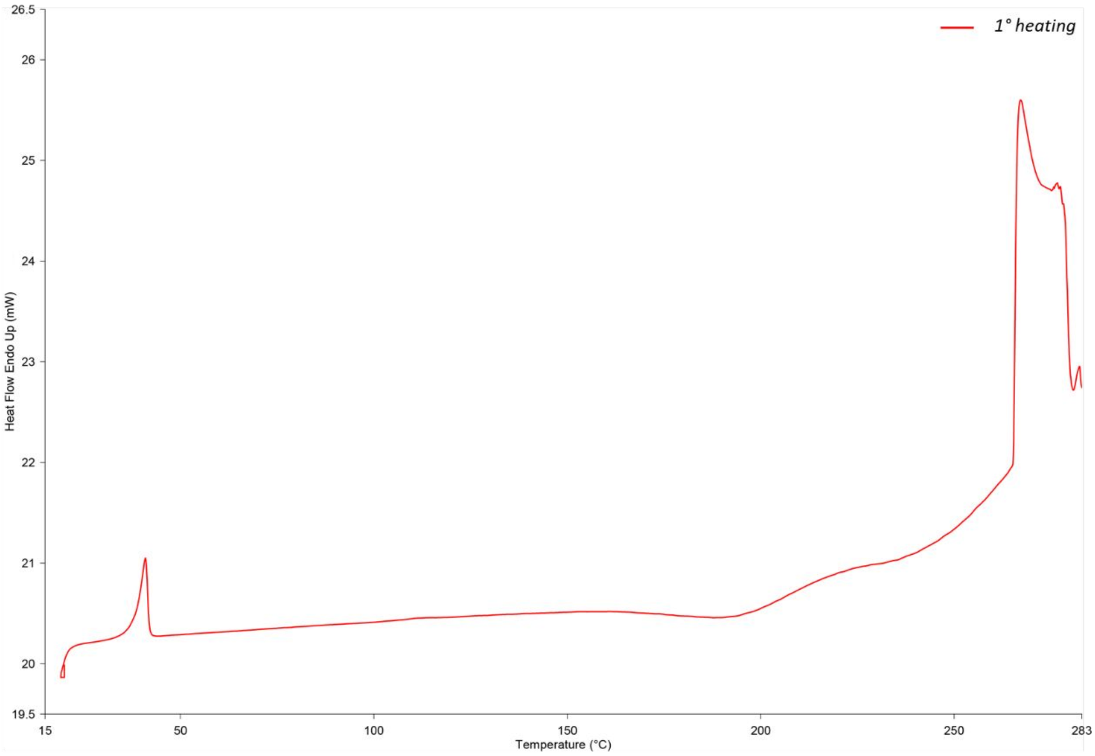

Figure SI 16. Thermogram of THY-HMT binary mixture with χ_{HMT} = 0.949 molar ratio. Heating run from 20 °C to 300 °C at 5 °C/min. No additional thermal treatments were performed due to since unusual endothermic peaks related to the decomposition of the sample.

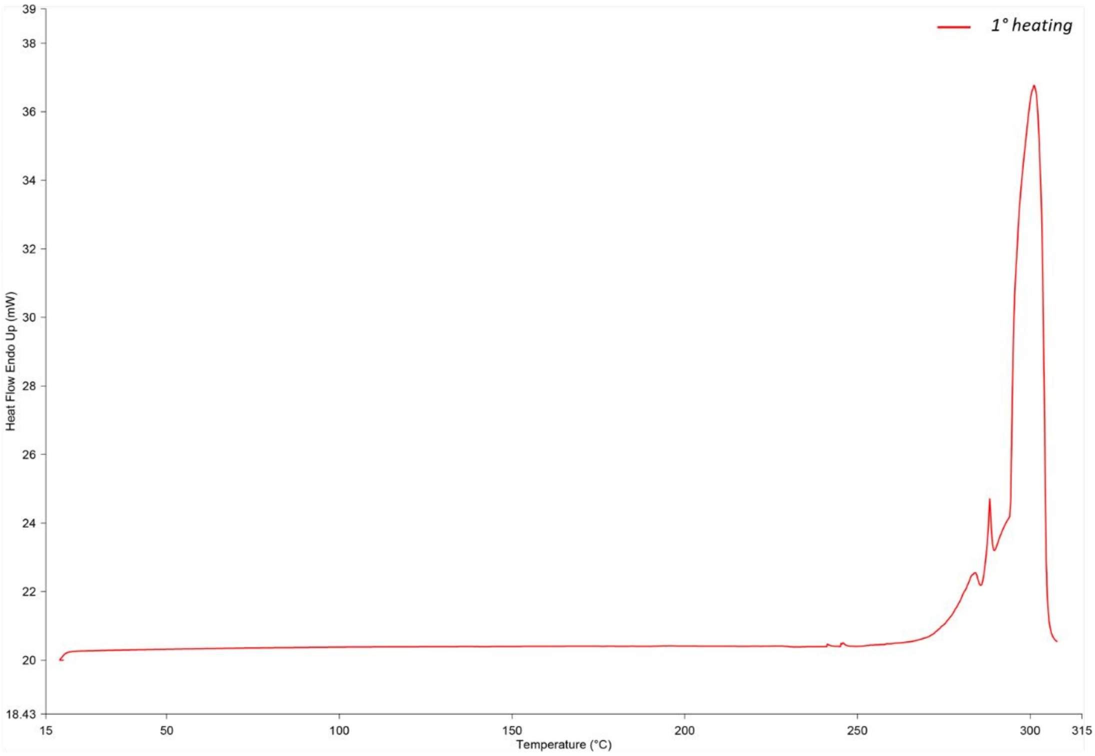

Figure SI 17. Thermogram of pure HMT. Heating run from 20 °C to 300 °C at 5 °C/min. No additional thermal treatments were performed due to since unusual endothermic peaks related to the decomposition of the sample.

Table SI 1. *Summary of thermal events occurred during DSC measurements for THY-HMT binary mixtures.*

THY-HMT molar ratio	Run		Thermal event	Temperature (°C)	ΔH (J/g)
$\chi_{HMT} = 0.000$ (THY)	First heating		Endothermic	47.94 [a]	135.84
	First cooling		Exothermic	-10.37 [b]	-90.44
	Second heating		Endothermic	47.69 [a]	133.92
$\chi_{HMT} = 0.026$	First heating	1st peak	Endothermic	26.21 [a]	10.64
		2nd peak	Endothermic	47.67 [b]	109.22
	First cooling		Exothermic	-1.97 [b]	-93.81
	Second heating	1st peak	Endothermic	23.57 [a]	7.37
		2nd peak	Endothermic	47.97	111.85
$\chi_{HMT} = 0.032$	First heating	1st peak	Endothermic	25.99 [a]	12.50
		2nd peak	Endothermic	47.73 [b]	105.00
	First cooling		Exothermic	-2.42 [b]	-91.77
	Second heating	1st peak	Endothermic	22.82 [a]	9.21
		2nd peak	Endothermic	47.89 [b]	105.17
$\chi_{HMT} = 0.048$	First heating	1st peak	Endothermic	26.43 [a]	19.97
		2nd peak	Endothermic	46.30 [b]	92.15
	First cooling	1st peak	Exothermic	-4.20 [b]	-84.08
	Second heating		Endothermic	23.38 [a]	15.11
		2nd peak	Endothermic	46.35 [b]	95.35
$\chi_{HMT} = 0.113$	First heating	1st peak	Endothermic	27.03 [a]	48.42
		2nd peak	Endothermic	35.81 [b]	34.65
	First cooling		--	--	--
	Second heating		--	--	--
$\chi_{HMT} = 0.131$	First heating	1st peak	Endothermic	27.27 [a]	58.56
		2nd peak	Endothermic	33.88 [b]	25.18
	First cooling		--	--	--
	Second heating		--	--	--
$\chi_{HMT} = 0.135$	First heating		Endothermic	27.35 [a]	87.83
	First cooling		--	--	--
	Second heating	1st peak	Exothermic	8.33 [b]	-29.58
		2nd peak	Endothermic	31.62 [b]	30.01
$\chi_{HMT} = 0.159$	First heating		Endothermic	26.75 [a]	77.82
	First cooling		--	--	--
	Second heating	1st peak	Exothermic	-3.20 [b]	-64.14
		2nd peak	Endothermic	31.77 [b]	71.30
$\chi_{HMT} = 0.192$	First heating	1st peak	Endothermic	26.99 [a]	37.91
		2nd peak	Endothermic	38.02 [b]	35.49
	First cooling		Exothermic	1.87 [b]	-20.27
		1st peak	Exothermic	10.83 [b]	-23.72
	Second heating	2nd peak	Endothermic	35.37 [b]	77.17
$\chi_{HMT} = 0.225$	First heating	1st peak	Endothermic	22.59 [a]	1.18
		2nd peak	Endothermic	41.70 [b]	56.31
	First cooling		Exothermic	10.77 [b]	-41.93
	Second heating		Endothermic	39.04 [b]	48.12
$\chi_{HMT} = 0.240$	First heating		Endothermic	40.79 [a]	66.61
	First cooling		Exothermic	8.50 [b]	-44.07
	Second heating		Endothermic	36.58 [b]	59.99
$\chi_{HMT} = 0.327$	First heating	1st peak	Endothermic	41.55 [a]	66.01
$\chi_{HMT} = 0.500$	First heating	1st peak	Endothermic	41.24 [a]	52.32
$\chi_{HMT} = 0.681$	First heating	1st peak	Endothermic	40.30 [a]	32.52
$\chi_{HMT} = 0.832$	First heating	1st peak	Endothermic	39.73 [a]	18.15
$\chi_{HMT} = 0.949$	First heating	1st peak	Endothermic	38.12 [a]	8.47
$\chi_{HMT} = 1.000$ (HMT)	First heating	1st peak	Endothermic	--	--

[a] onset temperature; [b] peak temperature

Binary phase diagram

The binary phase diagram was built reporting the experimental melting temperatures obtained by DSC measurements and the liquidus curve of THY, HMT and THY:HMT 3:1 cocrystal. The liquidus curve of THY was calculated using the Schroden-Laar law (Equation SI1, $R^2 = 0.909$) referred to a pure phase in a binary mixture. The liquidus curve of HMT was approximated to a linear fit due to its decomposition which preclude the determination of the melting temperatures for binary mixtures with an excess of HMT ($\chi_{HMT} > 0.25$). The liquidus curve of cocrystal was not calculated using a thermodynamic interpolation since no experimental points can be collected beyond the cocrystal composition ($\chi_{HMT} = 0.25$). Moreover, a thermodynamic fit requires at least three experimental temperatures (excluding those referring to the pure cocrystal and the eutectic composition) that would have all been grouped in a narrow range of molar compositions ($0.15 < \chi_{HMT} < 0.25$). Therefore, the liquidus curve of the cocrystal was calculated with a second-order mathematical fit (Equation SI2, $R^2 = 0.998$).

$$T_{calc}(°\mathrm{C}) = \left[T_{THY}\Delta H°_{THY}\left(\frac{1}{\Delta H°_{THY} - RT_{THY}\,ln\chi_{THY}}\right)\right] - 273.15 \qquad \text{Eq. SI1}$$

where $\Delta H°_{THY} = 6396.36\,J/mol$, $R = 8.31446\,J\,mol^{-1}K^{-1}$ and $T_{THY} = 323.48\,K$;

$$T_{calc}\,(°\mathrm{C}) = a(\chi_{HMT})^2 + b(\chi_{HMT}) + c \qquad \text{Eq. SI2}$$

where $a = 938.112$, $\mathrm{b} = -1962.540$ and $\mathrm{c} = -69.838$

Variable Temperature X-ray Powder Diffraction

VT-XRPD were performed on THY:HMT 3:1 cocrystal with a firing profile consisting of an heating (20°C-to-60°C) and cooling ramp (60°C-to-10°C), followed by a second heating run (10°C-to-60°C). The whole thermal treatment was performed at 5 °C/min.

The solid-state detector was used in 2D mode as reported in figure SI 17.

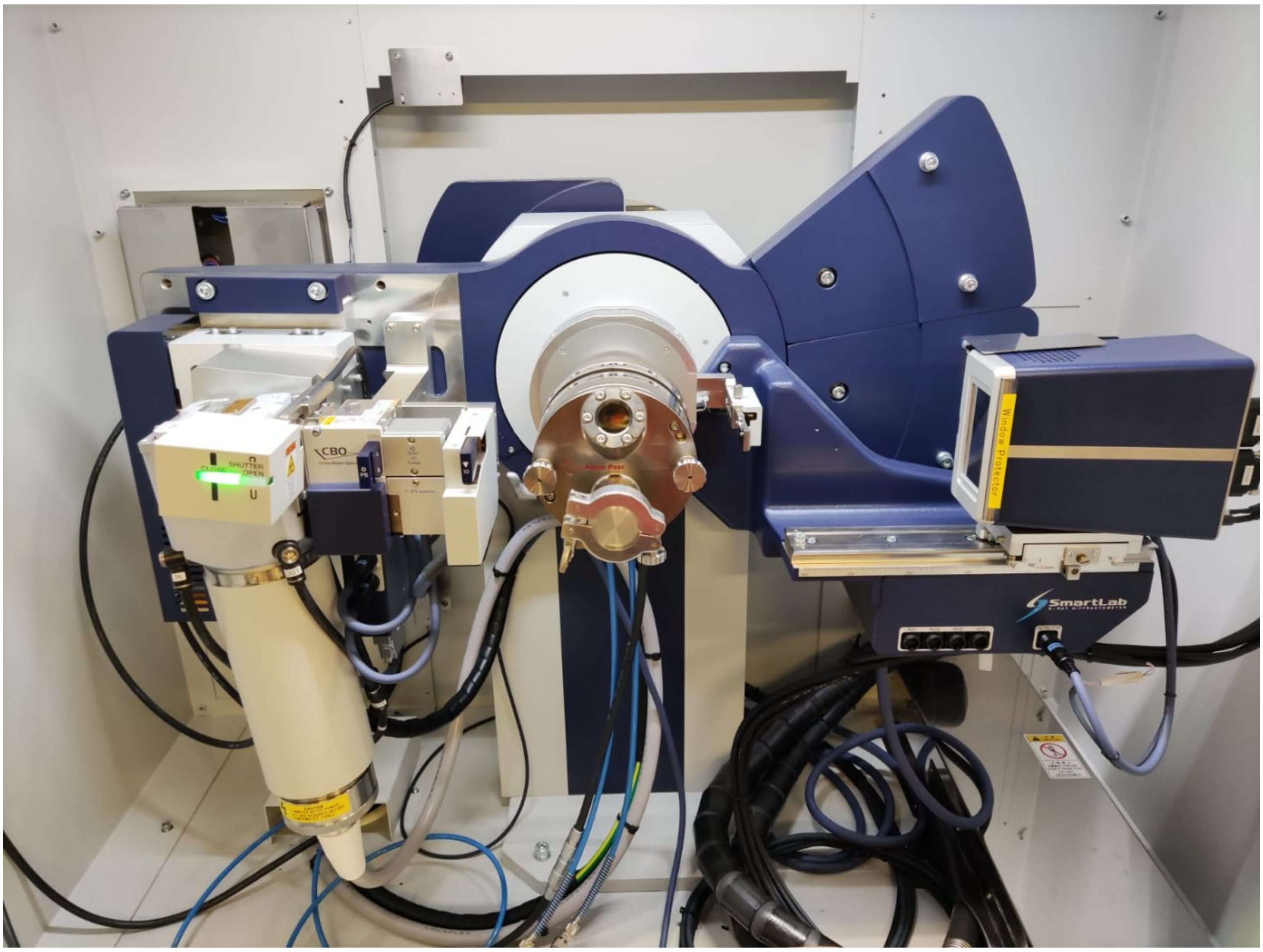

Figure SI 18. Picture of the setup, from left to right: Cu radiation source set at ω=4°, Non-ambient sample stage optimized in the vertical direction to minimize the sample displacement, 2D Hypix3000 SS detector placed at 300mm from the sample and set at 13° (2θ deg) thus covering the range $5<2\theta>19$.

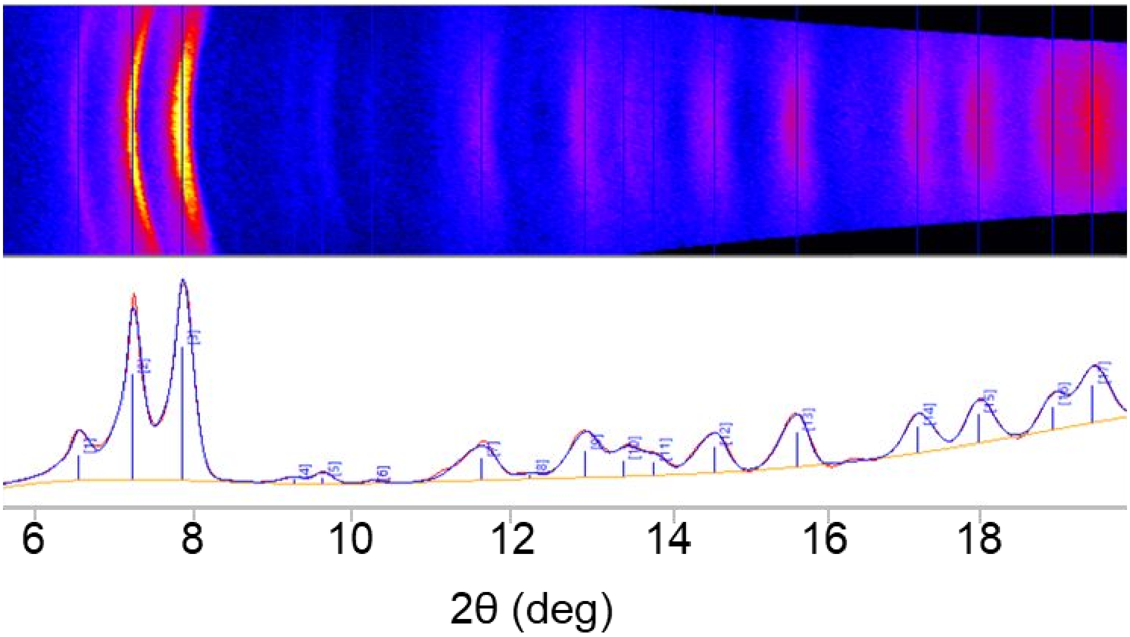

Figure SI 19. Up.2D-XRPD patter collected at the beginning of the firing profile. Down. Integrated XRPD pattern cut at (163°<β>197°) experimental data (red line), Every peak position (blue vertical lines) is calculated by fitting the experimental data with a Pseudo-Voight function (blue line). Background (yellow line).

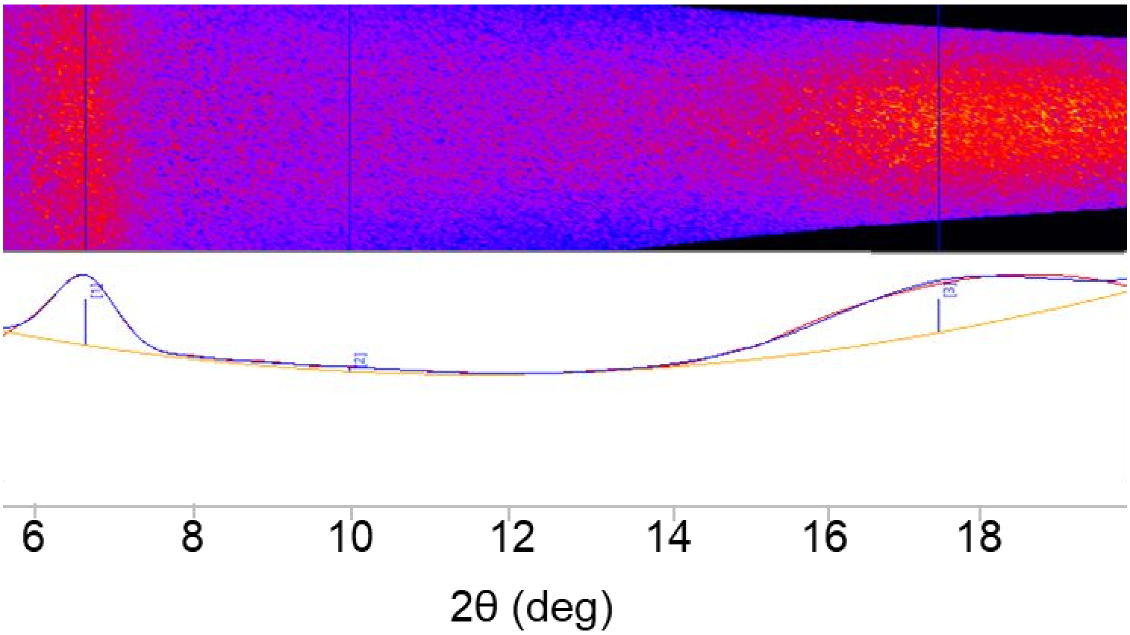

Figure SI 20. Up.2D-XRPD patter collected at 43°C during the first heating ramp. Down. Integrated XRPD pattern cut at (163°<β>197°) experimental data (red line), Every peak position (blue vertical lines) is calculated by fitting the experimental data with a Pseudo-Voight function (blue line). Background (yellow line).

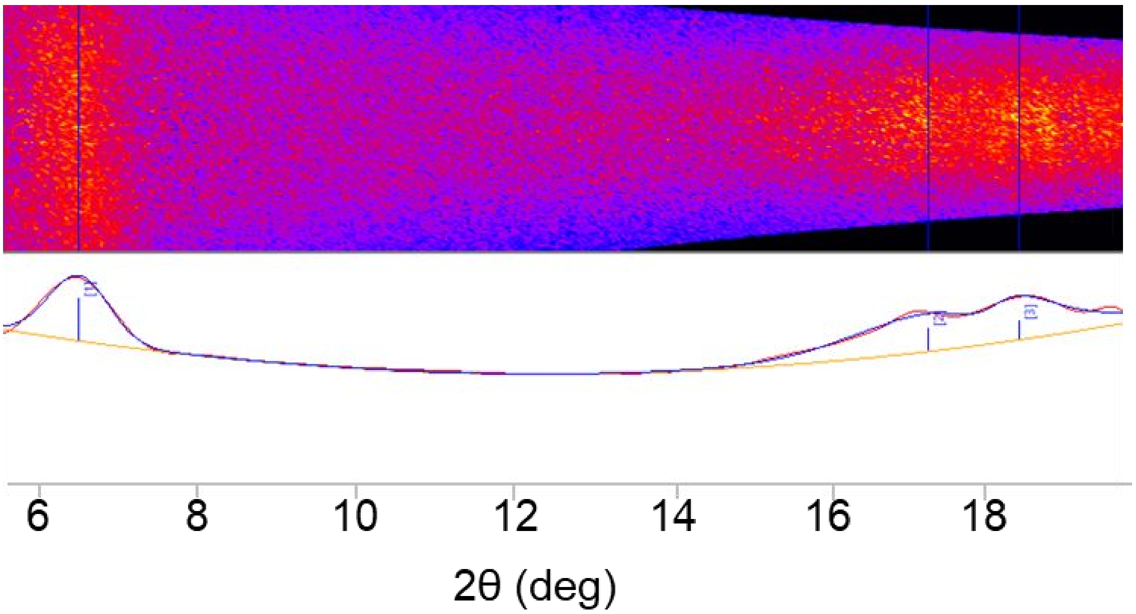

Figure SI 21. Up.2D-XRPD patter collected at 12°C during the first cooling ramp. Down. Integrated XRPD pattern cut at (163°<β>197°) experimental data (red line), Every peak position (blue vertical lines) is calculated by fitting the experimental data with a Pseudo-Voight function (blue line). Background (yellow line).

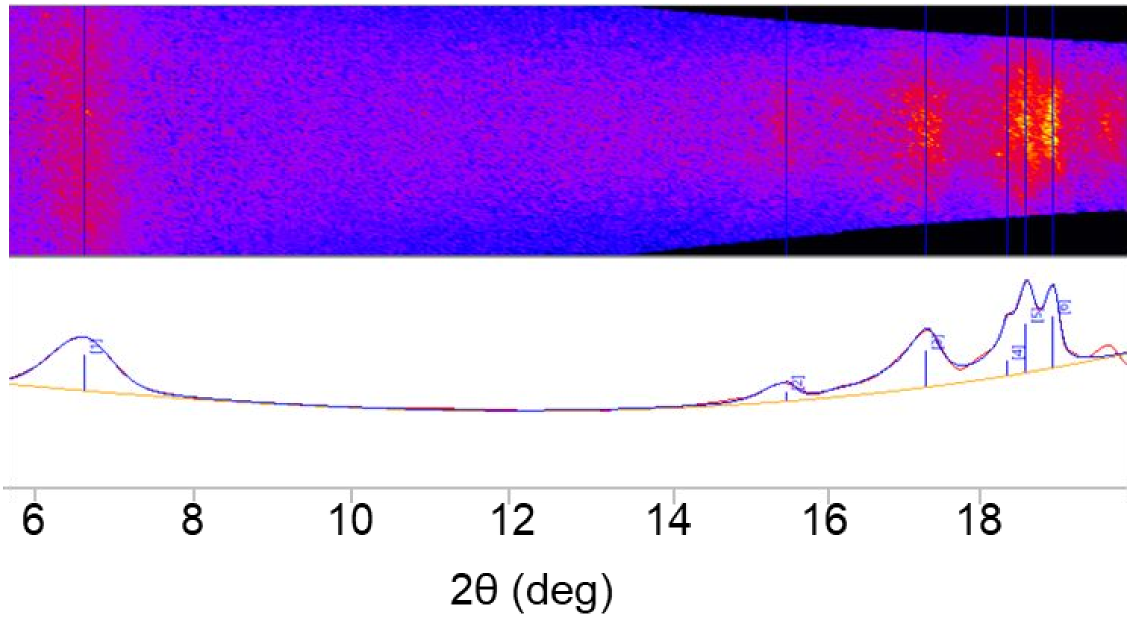

Figure SI 22. Up.2D-XRPD patter collected at 11°C during the first cooling ramp. Down. Integrated XRPD pattern cut at (163°<β>197°) experimental data (red line), Every peak position (blue vertical lines) is calculated by fitting the experimental data with a Pseudo-Voight function (blue line). Background (yellow line).

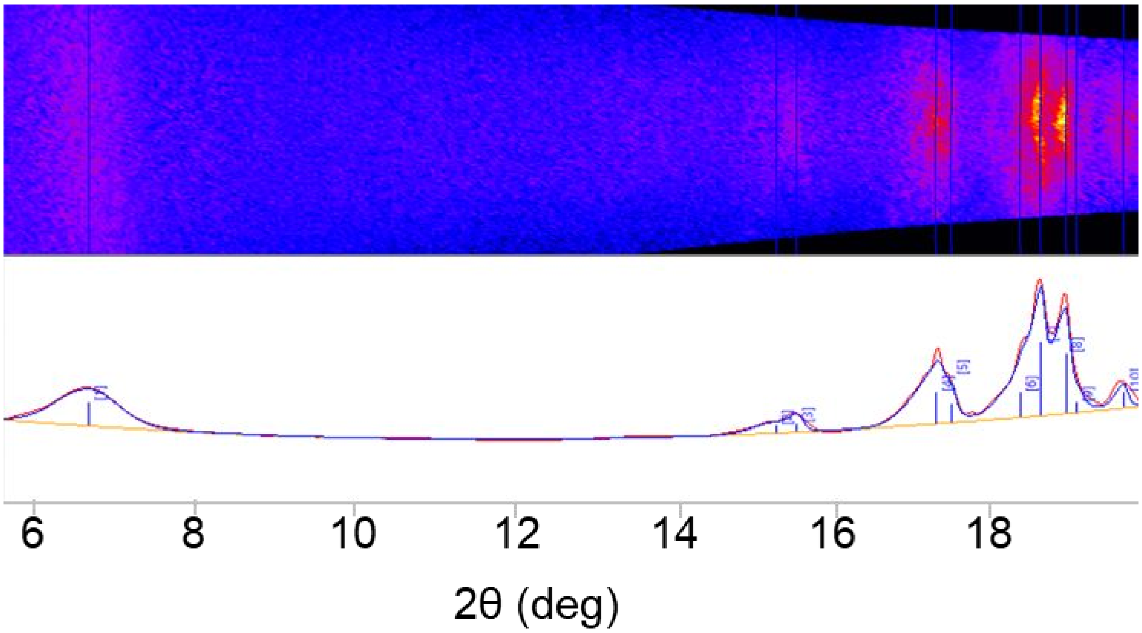

Figure SI 23. Up.2D-XRPD patter collected at 24°C during the second cooling ramp. Down. Integrated XRPD pattern cut at (163°<β>197°) experimental data (red line), Every peak position (blue vertical lines) is calculated by fitting the experimental data with a Pseudo-Voight function (blue line). Background (yellow line).

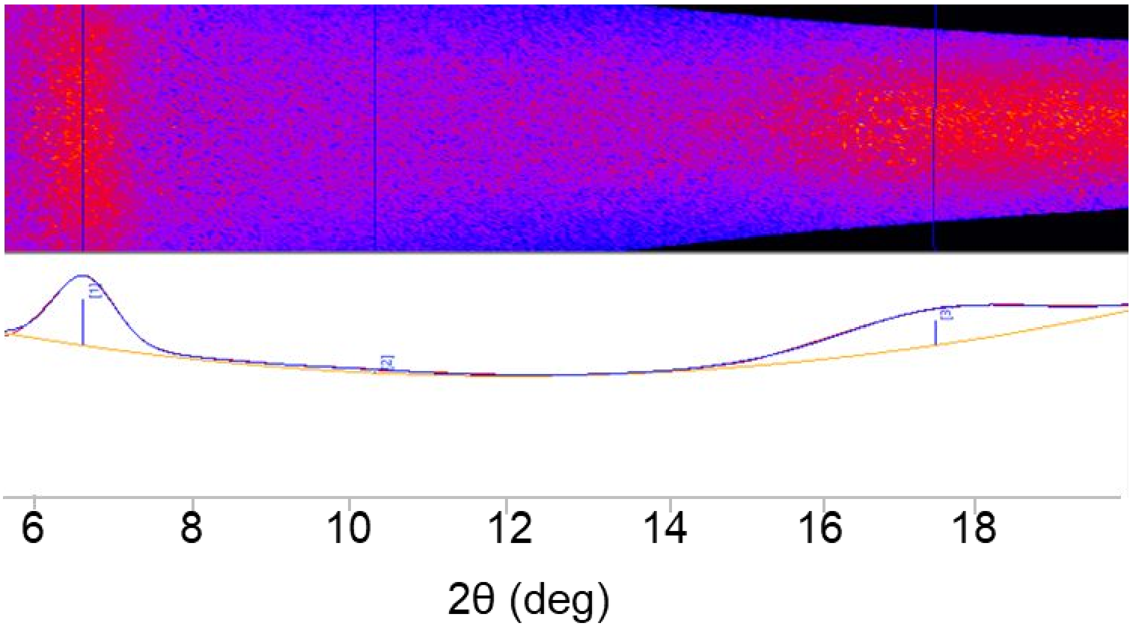

Figure SI 24. Up.2D-XRPD patter collected at 40°C during the second cooling ramp. Down. Integrated XRPD pattern cut at (163°<β>197°) experimental data (red line), Every peak position (blue vertical lines) is calculated by fitting the experimental data with a Pseudo-Voight function (blue line). Background (yellow line).

X-ray Powder Diffraction

High statistic XRPD pattern were collected in Bragg-Brentano geometry on the sample mounted in the non-ambient chamber. The 2D SS detector was used in 1D mode. 5° soller slits were used to improve peak shape and resolution.

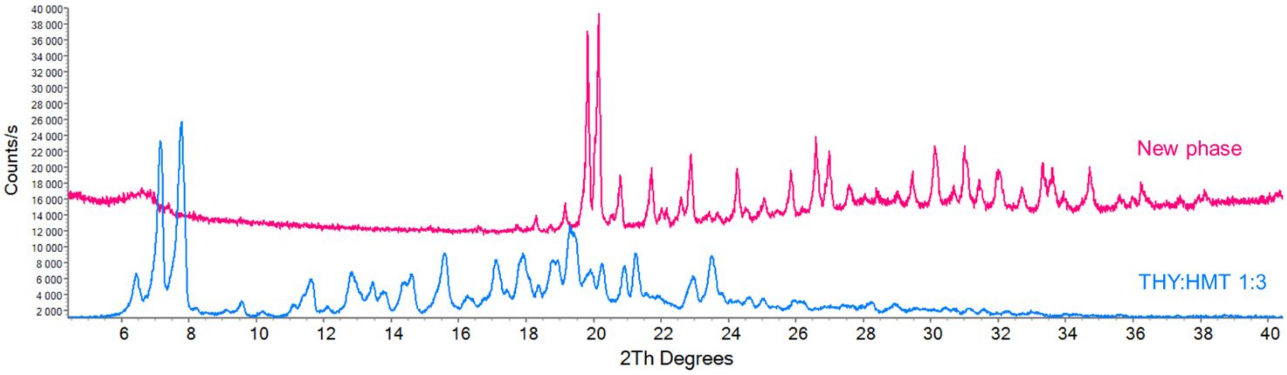

Figure SI 25. Experimental data collected in Brag-Brentano geometry at high statistic on THY:HMT 3:1 cocrystal (light blue line) superimposed to the experimental data collected after the thermal treatment showing a new crystalline phase (pink line).

Rietveld Refinements

Sequential Rietveld refinements were performed on data collected by TRIS-XRPD analysis to monitor the evolution of the mechanochemical synthesis of THY:HMT cocrystal. The strategy consisted in performing the Rietveld refinement of the pattern collected from the empty jar to derive the extrinsic background (figure SI26, blue line) that has been used in the sequential refinements of the samples under milling conditions.

The empty jar contribution to the background will only be scaled to be consistent with the baseline of the pattern under investigation. An additional amorphous phase, corresponding to the low melting eutectic phase, has been added as a peak phase to better fit the background (vertical blue lines represent the 2θ position of center of mass of the peaks for the peak phase). Thymol, HMT and cocrystal Bragg phases have been also refined against experimental data.

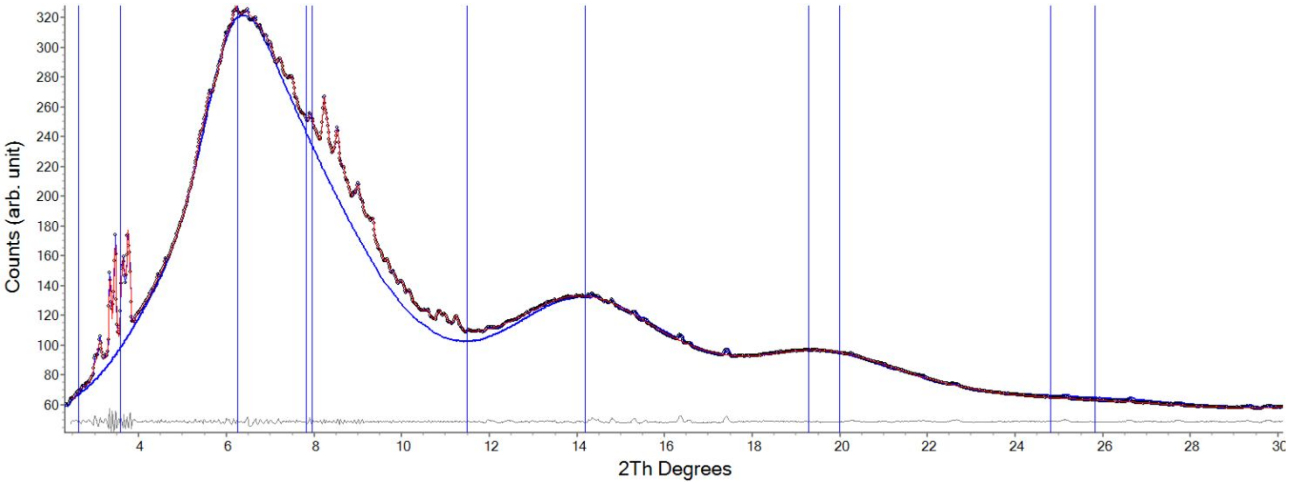

Figure SI 26. Rietveld refinement performed on XRPD pattern corresponding to 3 s of milling time. Calculated pattern (red line) against experimental data (black points). Differential Y_{cal}-Y_{obs} pattern is reported in grey. Blue curve represents the profile collected on the empty jar that contributes to the extrinsic background. Vertical lines represent the positions of the peak phase used to derive the intrinsic amorphous phase (*i.e.* LME) by difference from the extrinsic background and crystalline phases. Rwp = 0.685

Once all the structural and microstructural parameters have been defined for all phases, the scale factors were individually refined for every experimental patterns thus having a direct indication of the phase quantification. In a sequential Rietveld refinement strategy, the outputs are iteratively used as input for the following pattern.

Quantitative Phase Analysis outputs for THY, HMT, THY:HMT and LME are individually reported in figure SI 27. Sigmoidal fits were used to describe the evolution of reagents and products over time while the evaluation of the transient LME phase was fitted with a 5th order polynomial fit with a maximum observed at ca. 2 seconds. Statistical parameters of the fit are individually reported in the figure SI 27.

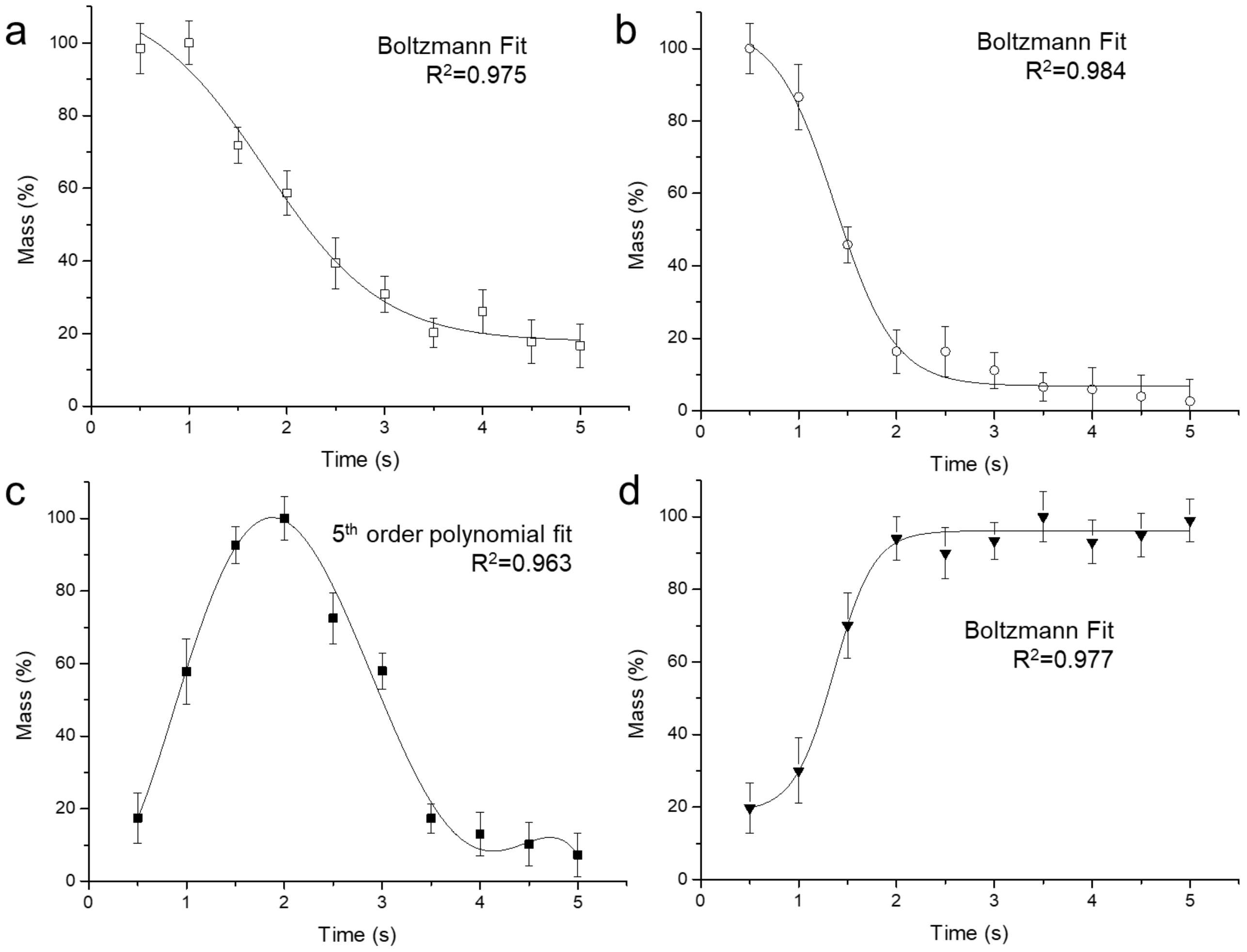

Figure SI 27. TRIS-XRPD Quantitative phase analysis evolution as a function of time for (a) Thymol, (b) HMT, (c) LME and (d) Cocrystal. Data were all fitted with a Boltzmann Sigmoidal fit except for the LME data that were fitted with a 5th order polynomial fit. Statistical parameters of the fit are reported for each graph.

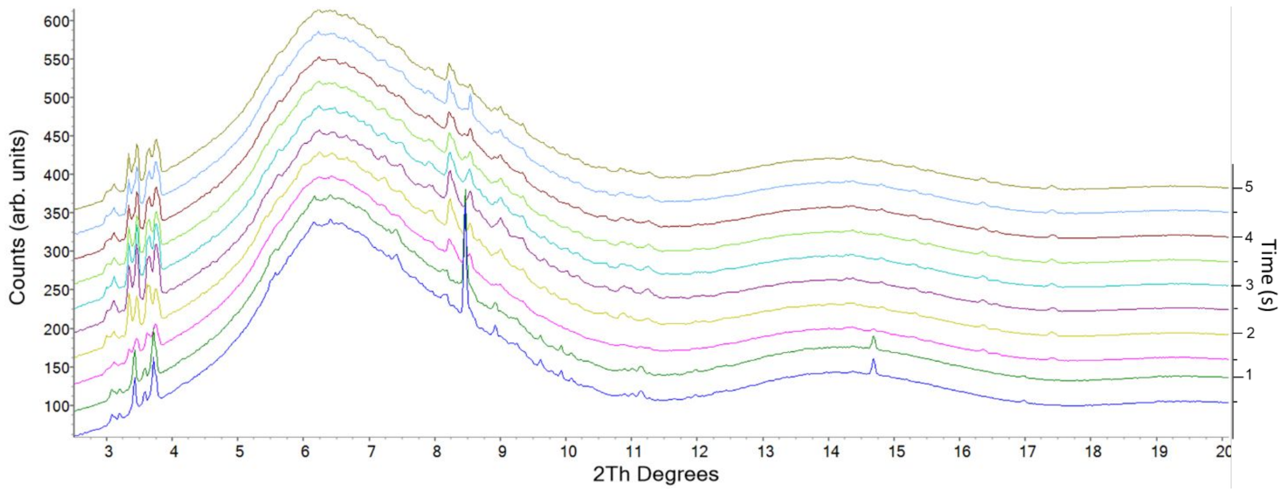

Figure SI 28. Cascade superimposition of TRIS-XRPD patterns collected as function of time.

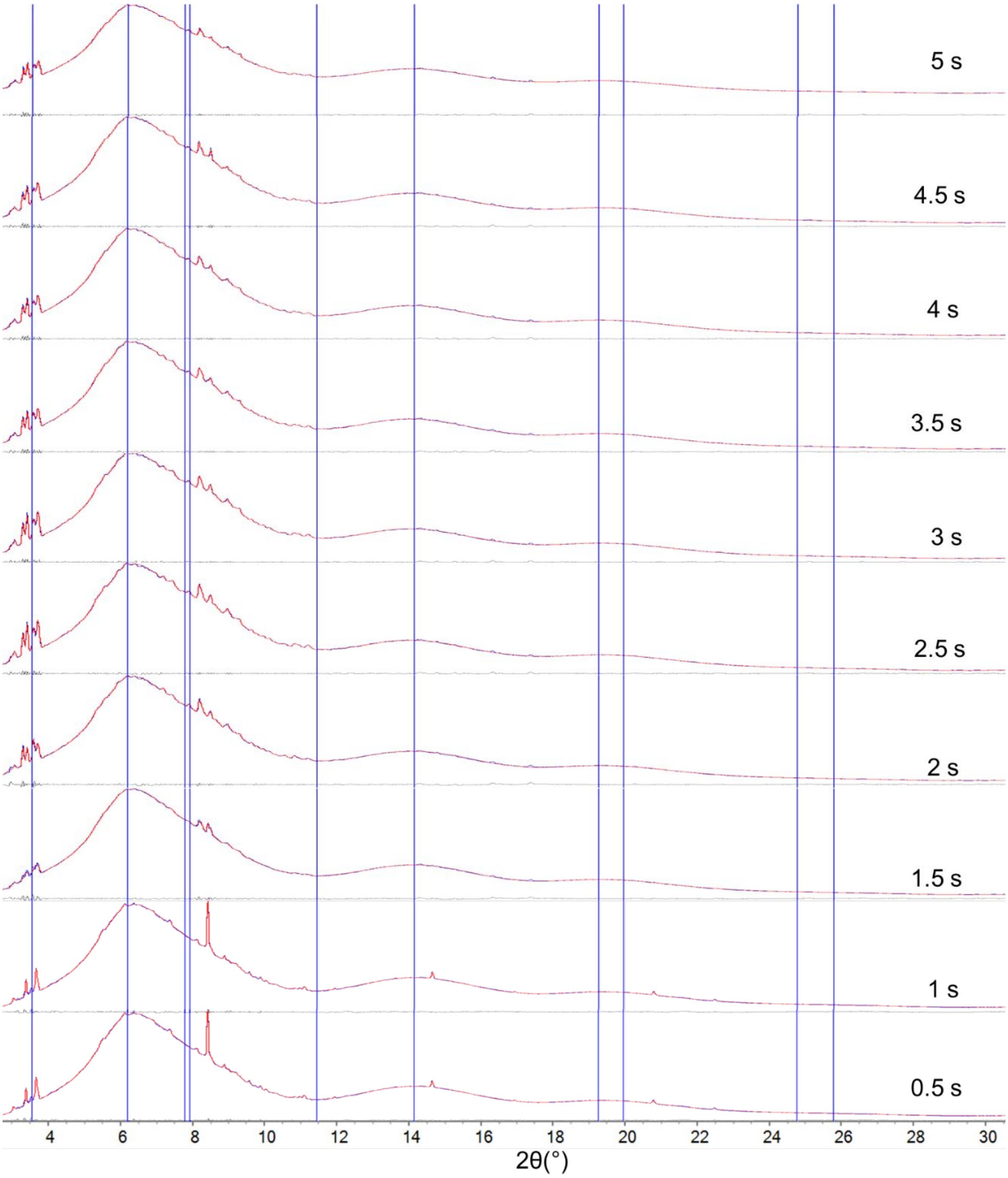

Figure SI 29. Rietveld refinement plot performed on XRPD patterns corresponding to 0.5 s – 5 s of milling time. Calculated pattern (red line) against experimental data (blue line). Differential Y_{cal}-Y_{obs} patterns are individually reported in grey. Vertical lines represent the positions of the peak phase used to derive the intrinsic amorphous phase (*i.e.* LME) and the extrinsic background contribution from the jar
